# Supplementary material for: Commonly used software tools produce conflicting and overly-optimistic AUPRC values
Source: Genome Biol. 2024 May 13;25:118. doi: 10.1186/s13059-024-03266-y (PMC11089773; doi:10.1186/s13059-024-03266-y)
Supplement: Supplementary file 1 — Additional file 1. Supplementary text, figures and tables. [file 13059_2024_3266_MOESM1_ESM.pdf]

Supplementary Text

Mathematical details of PRC and AUPRC

The non-linear interpolation formula

Suppose A is an anchor point and B is its next anchor point, defined by score thresholds t_A and t_B, respectively. In the most standard way to define anchor points, both t_A and t_B are classification scores of some entities, and t_B is the largest classification score of the entities that is smaller than t_A. If we predict all entities with a classification score $\geq t_A$ to belong to the positive class, there are TP_A true positives and FP_A false positives. Similarly, if we predict all entities with a classification score $\geq t_B$ to belong to the positive class, there are TP_B true positives and FP_B false positives, where $TP_B \geq TP_A$ and $FP_B \geq FP_A$. The value $m = (TP_B - TP_A) + (FP_B - FP_A)$ is the number of entities with a classification score of t_B. If $TP_B - TP_A > 1$, the interpolation methods interpolate additional point(s) between A and B.

One way to interpolate these additional points is to place them at their expected locations, assuming that all possible orders of the m entities have the same probability. Specifically, suppose at an additional point, there are $TP_A + tp$ true positives, where tp is an integer between 1 and $TP_B - TP_A - 1$. The expected number of false positives at this point is $FP_A + \frac{FP_B - FP_A}{TP_B - TP_A} tp$. As a result, the x and y coordinates of this additional point, which respectively correspond to its recall and precision, are:

$$\left(\frac{TP_A + tp}{P}, \frac{TP_A + tp}{TP_A + tp + FP_A + \frac{FP_B - FP_A}{TP_B - TP_A} tp} \right), \tag{1}$$

where P is the total number of actual positives.

The above method, proposed by Davis and Goadrich [2], interpolates $TP_B - TP_A - 1$ points with equal spacing along the x-axis between A and B. We refer to it as the "discrete expectation" method (Fig. 1h and Additional file 1: Fig. S3h). It should be noted that if we set tp to $TP_B - TP_A$, the formula produces the coordinates of anchor point B exactly. This is important for the continuous expectation method to be discussed below.

From Formula 1, it can be seen that precision does not necessarily change linearly with recall [2].

In the literature, some small variations of this method have been proposed. They also use Formula 1 to determine coordinates of the interpolated points but instead of placing $TP_B - TP_A - 1$ points with equal spacing along the x-axis between A and B, they place the points at fixed intervals of false positive count [33] or recall [20] by setting tp to corresponding (possibly non-integer) values accordingly. We call all these variations "discrete expectation" for simplicity. Also, some tools use the discrete expectation method to produce additional points even when there are no ties in classification scores (Fig. 1c and Additional file 1: Fig. S3c).

In Formula 1, if instead of considering just a finite number of discrete values of tp, it is allowed to take any real value between 0 and $TP_B - TP_A$, anchor points A and B will be connected by a continuous curve [33, 34]. We refer to this method as the "continuous expectation" method (Fig. 1i and Additional file 1: Fig. S3i). The area under the PRC between A and B can be computed by integrating the function defined by Formula 1:

$$\int_{\frac{TP_A}{P}}^{\frac{TP_B}{P}} \frac{TP_A + tp}{TP_A + tp + FP_A + \frac{FP_B - FP_A}{TP_B - TP_A} tp} \mathrm{d}r, \quad (2)$$

where $r = \frac{TP_A + tp}{P}$.

Let $r_1 = \frac{TP_A}{P}$, $r_2 = \frac{TP_B}{P}$, $h = \frac{FP_B - FP_A}{TP_B - TP_A}$, $a = 1 + h$, and $b = \frac{FP_A - hTP_A}{P}$, the integral in Formula 2 becomes [33]:

$$\begin{aligned} \int_{r_1}^{r_2} \frac{r}{ar + b} \mathrm{d}r &= \left. \frac{ar - b \ln(ar + b)}{a^2} \right|_{r=r_1}^{r=r_2} \\ &= \frac{ar_2 - b \ln(ar_2 + b) - ar_1 + b \ln(ar_1 + b)}{a^2} \end{aligned} \quad (3)$$

A previous study showed that practically, the discrete expectation and continuous expectation methods produce comparable results across various binary classification scenarios [33].

The Average Precision method

The Average Precision (AP) method [35] connects anchor points by step curves (Fig. 1e,j and Additional file 1: Fig. S3e,j). The AUPRC computed by the AP method represents the weighted mean of precision at different anchor points, where the weight is the increase in recall from the previous anchor point [15]:

$$AP = \sum_i (Recall_i - Recall_{i-1}) Precision_i \quad (4)$$

In the formula, the summation is taken over all anchor points i.

In information retrieval, where AP is widely applied, there is another well-accepted definition of AP. In this definition, instead of computing average precision at the anchor points defined by the unique classification scores of the entities, it computes average precision at a fixed number of points evenly spaced along the x-axis [36, 37, 38].

In computer vision, AP has become one of the most common assessment metrics in image segmentation. A recent study reported that five different definitions of AP coexist in bioimage segmentation analysis [39].

The tools we surveyed in the current study only implemented AP as defined by Formula 4 but not any of these other variations.

The starting point of PRC

In the main text, we defined the anchor points by the unique classification scores that the entities receive from the classifier. Specifically, for each unique score t, an anchor point is defined, which predicts all entities with a score $\geq t$ as positive and all other entities as not. If among the entities receiving the highest score at least one of them actually belongs to the positive class, the first anchor point will have a non-zero recall. In order for the PRC to cover the whole range of recall values from zero to one, we need a way to define a starting point that always has zero recall.

If the AP method is used to connect the starting point and the first anchor point, we can simply set the starting point to $(0, Precision_1)$, where $Precision_1$ is the precision of

the first anchor point. This is obvious if the first anchor point does not involve any entities actually coming from the positive class, in which case the recall of it would be zero and thus the starting point is identical to the first anchor point. On the other hand, if the first anchor point involves at least one entity actually coming from the positive class, by setting the precision of the starting point to be the same as the first anchor point, in the PRC these two points will be connected by a horizontal line, which matches the definition of AP in Formula 4.

If the continuous expectation method is used to connect the starting point and the first anchor point, we can use Formula 1 to determine the coordinates of the points on this connection curve. Substituting both TP_A and FP_A by 0, TP_B by TP_1 (number of true positives at the first anchor point), and FP_B by FP_1 (number of false positives at the first anchor point), the coordinates of the connection points become:

$$\begin{aligned} &\left(\frac{tp}{P}, \frac{tp}{tp + \frac{FP_1}{TP_1}tp}\right) \\ =&\left(\frac{tp}{P}, Precision_1\right) \end{aligned} \tag{5}$$

As tp approaches 0, the coordinates approach $(0, Precision_1)$.

Therefore, for both the AP method and the continuous expectation method, the starting point of the PRC is $(0, Precision_1)$.

Since the discrete expectation and continuous expectation methods both use the same way to determine locations of the interpolated points, we argue that when the discrete expectation method is used, it is reasonable to also set the starting of the PRC to $(0, Precision_1)$ following the continuous expectation method. In contrast, for the linear interpolation method, we were not able to find in the literature any proposed way to determine its starting point with a sound justification.

The baseline value of AUPRC

It is useful to benchmark the AUPRC value of a classifier against that of a baseline classifier. A commonly used baseline classifier is one that gives the same score to every entity. The corresponding PRC contains only one anchor point at $\left(1, \frac{P}{P+N}\right)$, where P and N are the actual numbers of positives and negatives, respectively.

If the AP method or the continuous expectation method is used to compute AUPRC, as mentioned above, the PRC of the baseline classifier will have a starting point of $\left(0, \frac{P}{P+N}\right)$, and thus the PRC is simply a horizontal line with an AUPRC of $\frac{P}{P+N}$. This quantity is called the baseline AUPRC value, which is simply the fraction of actual positives among all the entities [17, 40].

In the case of continuous expectation, we can also directly compute the AUPRC using Formula 3 by substituting TP_A and FP_A by 0, TP_B by P, and FP_B by N. Accordingly, the variables used in the formula will take the following values:

$$
\begin{aligned}
r_1 &= 0 \\
r_2 &= 1 \\
h &= \frac{N}{P} \\
a &= 1 + \frac{N}{P} \\
b &= 0
\end{aligned} \tag{6}
$$

Formula 3 then becomes:

$$
\begin{aligned}
&\frac{ar_2 - b\ln(ar_2 + b) - ar_1 + b\ln(ar_1 + b)}{a^2} \\
=&\frac{1 + \frac{N}{P} - 0 - 0 + 0}{(1 + \frac{N}{P})^2} \\
=&\frac{P}{P + N},
\end{aligned} \tag{7}
$$

which is the same as the value we obtained by considering the starting point of the PRC.

The five issues of deriving PRC and AUPRC

Description of the five issues

We found five issues when using the tools we analyzed to compute AUPRC values. These issues can be most easily explained by considering the corresponding PRC (Additional file 1: Fig. S4-S7), no matter the tools indeed first determine the points on the PRC and then compute the area under it accordingly, or rather compute AUPRC directly without first determining the points.

① Linear interpolation: When the linear interpolation method is used to handle ties in classification scores, theoretically the resulting AUPRC can be either larger (Fig. 1g-j) or smaller (Additional file 1: Fig. S3g-j) than the AUPRC computed by the other three methods.

Specifically, the linear interpolation method produces a larger AUPRC between the two anchor points than the AP method if the tie-associated anchor point (point B in Fig. 1g,j) has a smaller precision than the previous anchor point (point A in the figures). Conversely, the linear interpolation method produces a smaller AUPRC between the two anchor points than the AP method if the tie-associated anchor point has a larger precision than the previous anchor point (Additional file 1: Fig. S3g,j).

To compare the AUPRC values between the two anchor points produced by the linear interpolation and continuous expectation methods, we check whether the curve produced by the latter is convex or concave. Let $n = TP_A + tp + FP_A + \frac{FP_B - FP_A}{TP_B - TP_A} tp$, then according to Formula 1,

$$
\begin{aligned}
Precision &= \frac{TP_A + tp}{n} \\
\frac{dPrecision}{dtp} &= \frac{n - (TP_A + tp)\left(1 + \frac{FP_B - FP_A}{TP_B - TP_A}\right)}{n^2} \\
&= \frac{TP_B FP_A - TP_A FP_B}{(TP_B - TP_A)n^2} \\
\frac{d^2Precision}{dtp^2} &= -2\frac{TP_B FP_A - TP_A FP_B}{(TP_B - TP_A)n^3}\left(1 + \frac{FP_B - FP_A}{TP_B - TP_A}\right)
\end{aligned} \tag{8}
$$

Since $TP_B \geq TP_A$ and $FP_B \geq FP_A$, the sign of $\frac{d^2Precision}{dtp^2}$ depends only on $TP_B FP_A - TP_A FP_B$. There are three possibilities:

1. If $FP_B = FP_A = 0$, $\frac{d^2Precision}{dtp^2} = 0$. This means the continuous expectation method also connects the two anchor points by a straight line. Therefore the AUPRC between the two anchor points computed by the linear interpolation method is the same as the one computed by the continuous expectation method.

2. If $FP_A = 0$ but $FP_B \neq 0$, $\frac{d^2Precision}{dtp^2} > 0$. This means the continuous expectation method connects the two anchor points by a convex curve. Therefore the AURPC between the two anchor points computed by the linear interpolation method is larger than the one computed by the continuous expectation method.

3. If $FP_A \neq 0$ and $FP_B \neq 0$, $\frac{d^2Precision}{dtp^2} > 0 \Leftrightarrow \frac{TP_B}{FP_B} < \frac{TP_A}{FP_A}$. This means the continuous expectation method connects the two anchor points by i) a convex curve if the TP-to-FP ratio decreases from anchor point A to anchor point B, ii) a straight line if the TP-to-FP ratio does not change from A to B, and iii) a concave curve if the TP-to-FP ratio increases from A to B. Accordingly, the linear interpolation method produces a larger, equal, and smaller AUPRC between the two anchor points than the continuous expectation method, respectively.

This analysis also applies to the comparison between the linear interpolation method and the discrete expectation method, because the points interpolated by the discrete expectation method are all on the continuous curve interpolated by the continuous expectation method.

In practice, classifiers usually have a general decreasing trend of precision (and TP-to-FP ratio) as recall increases [41] because the entities receiving the highest classification scores are usually the ones that the classifier is most confident to be positives. Therefore, the linear interpolation method tends to be overly-optimistic as compared to the other three methods for connecting anchor points when multiple entities receive the same classification score.

② Using $(0, 1)$ as the starting point: As explained above, for both the continuous expectation and AP methods, the starting point of the PRC should be $(0, Precision_1)$, where $Precision_1$ is the precision of the first anchor point. When $Precision_1$ is not equal to one, setting the starting point to $(0, 1)$ will inflate the resulting AUPRC.

③ Incomplete PRC: The PRC is meant to show how the precision changes with recall over the whole range of possible values of recall (from 0 to 1). Some tools do not achieve it because they do not connect a point with zero recall to the first anchor point when the first anchor point has a non-zero recall.

④ Arbitrary tie breaking: When there are x entities with the same classification score, instead of generating one anchor point, x anchor points are generated as if the entities all have different classification scores. The order of these entities follow their input order. As a result, running a tool with such an issue multiple times with different input orders of these entities can produce different AUPRC values. Consequently, the ranking of different classifiers can be affected by the input order of the entities in ties, instead of depending on their performance alone.

⑤ Omitting anchor points: When there are multiple anchor points with the same recall, only the point with the highest precision is kept, which changes the resulting AUPRC value.

Consequences of the five issues

Among the five issues, four of them (①, ②, ④, and ⑤) can lead to overly-optimistic AUPRC values when there are ties in classification scores (Additional file 1: Table S2). Issue ⑤ (omitting anchor points) can also lead to overly-optimistic AUPRC values even when there are no ties in classification scores. Additional file 1: Fig. S8 provides some illustrative examples.

In addition, all five issues can change the order of two AUPRC values, which means a better-performing classifier can receive a lower AUPRC than a worse-performing classifier due to the issues (Additional file 1: Table S2 and Fig. S9-S13).

Detailed discussions of individual tools

ROCR

ROCR [14] is a tool in the R software environment for visualizing the performance of classifiers (Additional file 1: Table S1). We used the performance() function of ROCR to construct PRC and compute AUPRC. When there are no ties, ROCR connects adjacent anchor points by a straight line; When there are ties, it uses the discrete expectation method to connect adjacent anchor points to compute AUPRC (Table 1, Additional file 1: Fig. S4a, S5a, S6a, and S7a). Specifically, ROCR uses the expectation formula (Formula 1) to interpolate additional points with a step size of $tp = 1$ when two adjacent anchor points differs in their true positive counts by more than 2.

Regarding the calculation of AUPRC, we found that ROCR always starts the PRC at $(0, 1)$ (i.e., Issue ②). In addition, when there are consecutive anchor points with the same recall, ROCR retains only the one with the highest precision (i.e., Issue ⑤).

Regarding the PRC visualization, we found that ROCR only connects anchor points but not the starting point at zero recall (i.e., Issue II), which is inconsistent with the AUPRC value it produces.

In addition, we found a programming bug (⚠) in the source code of ROCR that can lead to AUPRC values larger than one. As an example, we ran ROCR on a data set using the following code:

```
score <- c(1,1,1,1,1,1,1,1,1,1,0,0,0,0)
label <- c(1,1,1,1,1,1,1,1,1,1,1,1,1,0)

# ROCR
pred <- prediction(score, label)
```

```
perf <- performance(pred, "aucpr")
print(perf@y.values)
```

ROCR returned an AUPRC value of 1.003. We found that the problem is caused by incorrect indices of the points on the PRC. Specifically, when additional points are interpolated between two anchor points, they are added to the list of points and therefore the indices of all points after them should be shifted. However, ROCR wrongly uses their original indices. The code from ROCR's GitHub repository (https://github.com/cran/ROCR/blob/master/R/performance_measures.R) is listed as follows:

```
for (i in seq_along(rec[-length(rec)])) {
  if (tp[i+1] - tp[i] > 2) {
    skew = (fp[i+1]-fp[i]) / (tp[i+1]-tp[i])
    x = seq(1, tp[i+1]-tp[i], by=1)
    rec <- append(rec, (x+tp[i])/n.pos, after=i)
    prec <- append(prec, (x+tp[i])/(tp[i]+fp[i]+x+ skew*x), after=i)
}
}
```

We fixed this bug by correcting the indices of the precision and recall lists after each interpolation:

```
j <- 0
for (i in seq_along(rec[-length(rec)])) {
  if (tp[i+1] - tp[i] > 2) {
    skew = (fp[i+1]-fp[i]) / (tp[i+1]-tp[i])
    x = seq(1, tp[i+1]-tp[i], by=1)
    rec <- append(rec, (x+tp[i])/n.pos, after=i+j)
    prec <- append(prec, (x+tp[i])/(tp[i]+fp[i]+x+ skew*x), after=i+j)
    j <- j + length(x)
}
}
```

After fixing the bug, the resulting AUPRC of the example is 0.991. In the main text, all results and discussions about ROCR are about this bug-fixed version unless otherwise stated. We did not make any additional changes in response to the other issues of ROCR.

Weka

Weka (Waikato Environment for Knowledge Analysis) [22] is a Java-based tool for machine learning and data mining (Additional file 1: Table S1). It provides AUPRC calculations and PRC visualization. We used the graphical user interface of Weka to construct PRC and compute AUPRC. Underlying, the former uses the getCurve() method of the weka.classifiers.evaluation.ThresholdCurve class, and the latter uses the getPRCArea() method of the same class. Weka uses the AP method to compute AUPRC (Table 1, Additional file 1: Fig. S4b, S5b, S6b, and S7b). In the PRC visualization, it only connects the anchor points and therefore does not start the PRC at zero recall when the first anchor point has a non-zero recall (i.e., Issue II).

scikit-learn

scikit-learn [15] is a Python library that provides efficient tools for machine learning and statistical modeling (Additional file 1: Table S1). It provides two options for computing

AUPRC (Table 1), by the direct straight line/linear interpolation method (Additional file 1: Fig. S4c, S5c, S6c, and S7c), and the AP method (Additional file 1: Fig. S4d, S5d, S6d, and S7d), respectively. For the first option, we used the precision_recall_curve() and auc() functions of the sklearn.metrics module to compute AUPRC. For the second option, we used the PrecisionRecallDisplay.from_predictions() function of the sklearn.metrics module to construct PRC and the average_precision_score() function of the same module to compute AUPRC.

For the first option, if the sklearn.metrics.precision_recall_curve() function is used to construct the PRC and then the sklearn.metrics.auc() function is used to compute AUPRC, the linear interpolation method is used (i.e., Issue ①) and the PRC always starts at $(0, 1)$ (i.e., Issue ②).

On the manual pages of scikit-learn (based on the version in August 2023), some cautionary notes are provided regarding its AUPRC calculations:

- On the manual page about its model evaluation modules (`https://scikit-learn.org/stable/modules/model_evaluation.html#precision-recall-f-measure-metrics`), it is mentioned that the linear interpolation method can lead to overly-optimistic AUPRC values: "References [Davis2006] and [Flach2015] describe why a linear interpolation of points on the precision-recall curve provides an overly-optimistic measure of classifier performance. This linear interpolation is used when computing area under the curve with the trapezoidal rule in auc." Despite this cautionary note, some recently published studies still used this method to compute AUPRC [42, 43, 44, 45, 46, 47].

- On the manual page about the AP method, it is mentioned that the resulting AUPRC is different from the one computed by connecting adjacent anchor points by direct straight lines: "This implementation is not interpolated and is different from computing the area under the precision-recall curve with the trapezoidal rule, which uses linear interpolation and can be too optimistic."

For the second option, the PRC visualization provided by scikit-learn always starts at $(0, 1)$ (i.e., Issue III). We also noticed that in earlier versions of scikit-learn (before version 0.19), the second option of scikit-learn uses direct straight lines to connect anchor points, which is inconsistent with the AP approach meant to be taken by this option.

PerfMeas

PerfMeas (Performance Measures) [16] is an R library for computing performance measures of classification results (Additional file 1: Table S1). It provides AUPRC calculations and PRC visualizations (Table 1, Additional file 1: Fig. S4e, S5e, S6e, and S7e). We used the precision.recall.curves.plot() function to construct PRC, and the precision.at.all.recall.levels() and trap.rule.integral() functions to compute AUPRC. PerfMeas uses direct straight lines to connect adjacent anchor points no matter whether there are ties in classification scores (i.e., Issue ①) or not.

In the calculation of AUPRC, PerfMeas only computes the area under the points it defines (i.e., Issue ③), including the anchor points that correspond to the unique classification scores and additional points produced by following the input order of entities with the same classification score and handling them as if there are no ties. When multiple entities have

the same classification score, PerfMeas breaks ties based on their input order, and therefore different runs of PerfMeas with different input orders of the entities can produce different AUPRC values (i.e., Issue ④). The PRC visualization also has these issues (i.e., Issue I).

PRROC

PRROC [17] is an R package specializing in constructing the ROC and PRC and computing the areas under them (Additional file 1: Table S1). PRROC provides two options (Table 1). The first option uses straight lines to connect adjacent anchor points when there are no ties and the discrete expectation method for handling ties (Additional file 1: Fig. S4f, S5f, S6f, and S7f). The second option uses continuous expectation method no matter whether there are ties or not (Additional file 1: Fig. S4g, S5g, S6g, and S7g). For both options, we used the pr.curve() function to construct the PRC and compute AUPRC, although with different parameter values to specify the options, and the plot() function to visualize the PRC.

PRROC constructs PRC by defining a finite number of points on it. When there are more than 100 actual positives, the PRCs constructed by the two methods are identical, both based on Formula 1 with tp set to 1. Otherwise, the continuous expectation method of PRROC places points at regular intervals along the x-axis, with an increase of 0.01 recall between every two adjacent points. When using the continuous expectation method, PRROC computes the AUPRC directly using Formula 3 rather than computing the area under the PRC it constructs.

We discovered a programming bug (⚠) in the visualization of PRC when the discrete expectation method is used to handle ties. The bug is due to the inexact nature of floating-point number computation, which creates errors in sorting (Additional file 1: Fig. S20). The part of the code that contains this bug is not used in the calculation of AUPRC, and therefore the correctness of AUPRC is not affected by it.

TensorFlow

TensorFlow [21] is a machine learning platform in Python and C++, most commonly used for deep learning (Additional file 1: Table S1). We used the tensorflow.keras.metrics.AUC() function to compute AUPRC. It computes AUPRC using the continuous expectation method without providing PRC visualizations (Table 1). We conceptually derive the AUPRC that corresponds to the AUPRC computed (Additional file 1: Fig. S4h, S5h, S6h, and S7h).

We give three cautionary notes (⚠) about using TensorFlow to compute AURPC. First, unlike most other tools, which use each unique classification score as a threshold, TensorFlow by default uses a user parameter (default to 200) to define the number of thresholds, which are then distributed evenly between zero and one. This uniform sampling approach is most accurate when the classification scores are distributed uniformly between zero and one, and therefore it is not suitable when most classification scores are concentrated in a small range of values. In addition, if the user parameter is set to a small value, the resulting AUPRC can deviate substantially from one computed by using each unique classification score as a threshold. Alternatively, TensorFlow provides an option for the user to provide a list of thresholds to use.

When different numbers of thresholds are used or when different threshold lists are provided, the resulting AUPRC computed can be different. We illustrate this issue in Additional

file 1: Fig. S21 based on an example provided on the manual page of TensorFlow that explains how AUPRC can be computed (https://tensorflow.google.cn/api_docs/python/tf/keras/metrics/AUC).

Second, due to the first cautionary note above, TensorFlow expects all classification scores to be between zero and one. Classification scores outside this range will not create a run time error, but they can make the AUPRC calculated inaccurate.

Third, instead of considering all entities with a classification score larger than or equal to the threshold as positive, which is what we described in the main text and implemented by most tools, TensorFlow considers only entities with a classification score larger than the threshold as positive (the smallest threshold that TensorFlow defines is a small negative value, such that entities with a zero classification score are all included in the last anchor point). We could not find any warnings about this cautionary notes on the official web site of TensorFlow.

precrec

precrec [20] is an R package specializing in constructing the ROC and PRC and computing the areas under them (Additional file 1: Table S1). We used the evalmod() function to compute AUPRC.

precrec uses the discrete expectation method to compute AUPRC (Table 1, Additional file 1: Fig. S4i, S5i, S6i, and S7i). When there are no ties in classification scores, it creates points on the PRC in regular intervals of recall, where the length of each interval is a user parameter (default to 0.001), and computes the precision of these points using the expectation formula. When there are x entities with the same classification score, it first interpolates TP and FP to generate x points in total, converts them into recall and precision, and then creates points on the PRC in regular intervals of recall in the same way as it does when there are no ties.

TorchEval

TorchEval [23] is a library of the PyTorch machine learning framework, most commonly used in deep learning, for evaluating model performance (Additional file 1: Table S1). We used the tourcheval.metrics.BinaryAUPRC() function to compute AUPRC. It uses the AP method to compute AUPRC without providing PRC visualizations (Table 1). We conceptually derive the AUPRC that corresponds to the AUPRC computed (Additional file 1: Fig. S4j, S5j, S6j, and S7j).

We did not find any issues with the AUPRC calculation of TorchEval.

MLeval

MLeval [18] is an R package for evaluating machine learning models (Additional file 1: Table S1). We used the evalm() function to construct PRC and compute AUPRC. It uses straight lines to connect adjacent anchor points when there are no ties and the linear interpolation method for handling ties (i.e., Issue ①) (Table 1, Additional file 1: Fig. S4k, S5k, S6k, and S7k). It only connects anchor points, which means the PRC does not cover the area with a recall values smaller than that of the first anchor point (i.e., Issue ③). The PRC visualization has the same issues (i.e., Issue I).

yardstick

yardstick [19] is an R package that provides various metrics for quantifying how well a model fits a data set (Additional file 1: Table S1). It provides two options for computing AUPRC (Table 1), by the direct straight line/linear interpolation method (Additional file 1: Fig. S4l, S5l, S6l, and S7l) and the AP method (Additional file 1: Fig. S4m, S5m, S6m, and S7m), respectively. For the first option, we used the pr_curve() function to construct PRC and the pr_auc() function to compute AUPRC. For the second option, we used the average_precision() function to compute AUPRC.

For the first option, in addition to the issue of linear interpolation (i.e., Issue ①), yardstick also always starts the PRC at $(0, 1)$ (i.e., Issue ②). The PRC visualization has the same issues (i.e., Issue I).

The ROC and AUROC

Although this study focuses on issues related to PRC and AUPRC, all the tools for producing PRC and AUPRC we analyzed can also compute AUROC. We also found some tools that can compute AUROC but not AUPRC (Additional file 1: Table S1). Since ROC and PRC are conceptually highly related to each other and practically both commonly used, here we also explain the concepts behind ROC and AUROC and their implementations.

Definition of the ROC and AUROC

The ROC plots the true positive rate (TPR) against the false positive rate (FPR) (Additional file 1: Fig. S1). In the most common way to produce the ROC, which is used by all the 12 tools we analyzed (Additional file 1: Fig. S1), each unique classification score is used as a threshold to define an anchor point of the ROC. When no two entities have the same classification score, two adjacent anchor points are connected directly by a straight line. The starting point of the ROC is obvious from its definition: if a threshold larger than the classification scores of all the entities is used, none of the entities is predicted to belong to the target class. In that situation, both the TPR and the FPR are zero. Therefore, the ROC always starts at $(0, 0)$.

When multiple entities have the same classification score, they together define a single anchor point. This anchor point is connected to the previous anchor point by a direct straight line, assuming that all possible orders of these entities are equally likely. In other words, ties are handled by taking the expectation [48] (Additional file 1: Fig. S22). To understand why the expectation curve is linear in the case of ROC but non-linear in the case of PRC, we note that in the definitions of TPR and FPR, the denominator is a constant (total number of actual positives and total number of actual negatives, respectively) and therefore TPR is directly proportional to the number of true positives so far and FPR is directly proportional to the number of false positives so far. In contrast, in the definition of precision, the denominator is the total number of true positives and false positives so far. Therefore, precision is not directly proportional to the number of true positives.

All the tools we surveyed handled ties in this way. The only difference between them is whether additional points are interpolated between the anchor points. These additional points do not change the shape of the ROC or the calculation of AUROC, but they can be

useful for computing confidence intervals [20].

A recent study argued that when there are ties, directly connecting adjacent anchor points by straight lines is not appropriate when the classifier produces discrete predictions [49]. This is still under debate.

Algorithms for plotting the ROC

There are two commonly used algorithms for plotting the ROC, which produce identical results but differ from each other slightly in terms of their implementations.

The first algorithm considers the segment of the ROC directly caused by each entity one by one when there are no ties. Specifically, all entities are first sorted in descending order of their classification scores. Then each entity on the sorted list is visited sequentially. If it is a true positive, the ROC goes up vertically by $\frac{1}{P}$, where P is the total number of actual positives among all the entities. If it is a false positive, the ROC goes right horizontally by $\frac{1}{N}$, where N is the total number of actual negatives among all the entities. When multiple entities have the same classification score, the previous anchor point is connected to a new anchor point that has an increase of $\frac{tp}{P}$ TPR and an increase of $\frac{fp}{P}$ FPR, where tp and fp are the number of actual positives and actual negatives among these entities (Additional file 1: Fig. S23a, b).

The second algorithm uses the definition of ROC directly. It computes the coordinates of each anchor point based on the TPR and FPR when its threshold is used to predict entities that belong to the positive class (Additional file 1: Fig. S23a, c). To determine the anchor points, most of the tools also sort the entities in descending order of their classification scores.

Algorithms for computing AUROC

There are two common algorithms for computing AUROC, which produce identical results [50, 51].

The first one accumulates the change of AUROC by summing up the areas of all the trapezoids (Additional file 1: Fig. S24). Each accumulation happens either when an anchor point has a FPR larger than the previous anchor point (the vertical trapezoidal rule, Additional file 1: Fig. S24a) or when an anchor point has a TPR larger than the previous anchor point (the horizontal trapezoidal rule, Additional file 1: Fig. S24b). Most of the tools we surveyed use the vertical trapezoidal rule, including ROCR, pROC [52], scikit-learn, PRROC, TensorFlow, plotROC [53], precrec (default mode), TorchEval, MLeval, and yardstick (Additional file 1: Table S3). Weka uses the horizontal trapezoidal rule and further divides each trapezoid into a triangle and a rectangle.

The second method computes the Wilcoxon-Mann-Whitney statistic and then converts it to AUROC (Additional file 1: Fig. S25, to be explained in the next section below). Precrec (aucroc mode) and PerfMeas compute AUROC in this way with slightly different procedures.

There are some other metrics that are either closely related to the Wilcoxon-Mann-Whitney statistic or identical to it under certain circumstances. These metrics can also be used to compute AUROC. For example, the concordance index, which is an evaluation metric frequently used in survival analyses, is identical to the Wilcoxon-Mann-Whitney statistic when predictions are binary [54].

Relationship between the Wilcoxon-Mann-Whitney statistic and AUROC

Suppose there are two lists of real values. The Wilcoxon-Mann-Whitney statistic quantifies the difference between them based on the sum of the ranks of the values from each list when the two lists are merged. Conceptually, if the two lists have comparable values, their rank sums should be similar. Using this statistic, the Wilcoxon-Mann-Whitney test (also called "rank-sum test" and "U test") compares two distributions based on a sample from each of them (i.e., the two lists), under the null assumption that a value randomly sampled from the first distribution has equal chance to be larger than or smaller than a value randomly sampled from the second distribution. By considering only the ranks but not the original values, the Wilcoxon-Mann-Whitney test is not specific to any parametric family of distributions.

Mathematically, the Wilcoxon-Mann-Whitney statistic based on the values on the first list is defined as

$$U_1 = \sum_{i=1}^{n_1} r_i - \frac{n_1(n_1+1)}{2}, \tag{9}$$

where n_1 is the number of values on the first list and r_i is the rank of its i-th largest value in the merged list (largest value in the merged list has rank 1). Notice that if the merged list contains duplicated values, they all share the same average rank. For example, if the four largest values on the merged list are the same, they all have a rank of $\frac{1+2+3+4}{4} = 2.5$.

According to the definition, the Wilcoxon-Mann-Whitney statistic is the difference between two components. The first component is the rank sum of the values in the merged list. The second component is the smallest possible rank sum of n_1 values in the merged list, which happens when all values on the first list are larger than all values on the second list. The Wilcoxon-Mann-Whitney statistic therefore quantifies how far the actual rank sum is from the minimum.

To see how U_1 is related to AUROC, we treat the first list as the classification scores of the actual positive entities and the second list as the classification scores of the actual negative entities. Therefore, $n_1 = P$. For simplicity of notations and without loss of generality, we assume that both lists are sorted in descending order.

First, we consider the situation that all entities have unique classification scores. The contribution of the i-th entity on the actual positive list to the AUROC is the area of a rectangular stripe with a height of one unit and a width of $N - f_i$ units, where f_i is the number of actual negatives having a higher classification score than the i-th actual positive entity (Additional file 1: Fig. S25a). After normalizing by the total number of actual positives and total number of actual negatives, the area of this rectangle is:

$$\frac{N - f_i}{N \times P} \tag{10}$$

Summing over the contributions of all the actual positive entities, we get the AUROC:

$$\begin{aligned} AUROC &= \sum_{i=1}^{P} \frac{N - f_i}{N \times P} \\ &= 1 - \frac{1}{N \times P} \sum_{i=1}^{P} f_i \end{aligned} \tag{11}$$

Now, the rank of an actual positive entity in the merged list is equal to the number of entities with a classification score higher than or equal to it, including itself, other actual positive entities, and actual negative entities. Therefore, $r_i = i + f_i$. This means the formula of AUROC can be re-written as follows:

$$
\begin{aligned}
AUROC &= 1 - \frac{1}{N \times P} \sum_{i=1}^{P} (r_i - i) \\
&= 1 - \frac{(\sum_{i=1}^{P} r_i) - P(P+1)/2}{N \times P} \\
&= 1 - \frac{U_1}{N \times P},
\end{aligned}
\tag{12}
$$

which provides a way to compute AUROC from the Wilcoxon-Mann-Whitney statistic [55].

Next, we consider the situation that some entities have the same classification score. Suppose there is a group of entities that have the same classification score, among which there are p actual positives and n actual negatives. Suppose also that in the list of actual positives and the merged list of actual positives and actual negatives (both lists sorted in descending order of classification scores), the first entry with this classification score is the i-th entry and the r_i-th entry, respectively. As mentioned above, each entity in this group has an average rank of $\tilde{r_i} = \frac{r_i + (r_i + p + n - 1)}{2} = r_i + \frac{p+n-1}{2}$ in the merged list. The total contribution of this group to the AUROC is the area of a trapezoid with a height of p units, a width of $N - f_i$ on the lower, longer side, and a width of $N - f_i - n$ on the upper, shorter side (Additional file 1: Fig. S25b). After normalizing by the total number of actual positives and total number of actual negatives, the area of this trapezoid is:

$$
\begin{aligned}
&\frac{p[(N - f_i) + (N - f_i - n)]/2}{N \times P} \\
=&\frac{p[N - (f_i + n/2)]}{N \times P} \\
=&\frac{p[N - (r_i - i + n/2)]}{N \times P} \\
=&\frac{p\{N - [r_i + (p + n - 1)/2 - (2i + p - 1)/2)]\}}{N \times P} \\
=&\frac{p\{N - [\tilde{r_i} - (2i + p - 1)/2)]\}}{N \times P} \\
=&\frac{\sum_{j=i}^{i+p-1} [N - (\tilde{r_i} - j)]}{N \times P},
\end{aligned}
\tag{13}
$$

which has the same form as the summation formula for computing the AUROC based on the contributions of individual actual positive entities (Formula 11). Therefore, AUROC can still be computed from the Wilcoxon-Mann-Whitney statistic using Formula 12 when all entities in ties are given their average rank.

Special notes on the implementations of AUROC calculation by different tools

Precrec (aucroc mode) computes AUROC from the Wilcoxon-Mann-Whitney statistic essentially following our description above, except that it sorts classification scores in ascending order and adjusts the formulas accordingly.

PerfMeas also computes AUROC from the Wilcoxon-Mann-Whitney statistic essentially following our description above, except that it sums over contributions of individual actual negatives and adjusts the formulas accordingly.

TensorFlow's AUROC calculation also uses the designs that we discussed in the three cautionary notes for its AUPRC calculation. Therefore, the accuracy of the AUROC computed also depends on the distribution of classification scores. In the same official example provided by TensorFlow (Additional file 1: Fig. S21), the correct AUROC computed by having 3 thresholds seems only a coincidence.

Supplementary Figures

Predicted Class		Actual Class: Positive (P)	Actual Class: Negative (N)
	Positive	True Positive (TP)	False Positive (FP)
	Negative	False Negative (FN)	True Negative (TN)

$$\text{True Positive Rate (TPR)} = \frac{TP}{TP+FN} = \frac{TP}{P}$$

$$\text{False Positive Rate (FPR)} = \frac{FP}{FP+TN} = \frac{FP}{N}$$

$$\text{Accuracy (ACC)} = \frac{TP+TN}{TP+TN+FP+FN} = \frac{TP+TN}{P+N}$$

$$F_1 = \frac{2TP}{2TP+FP+FN} = 2\times\frac{\text{Precision}\times TPR}{\text{Precision}+TPR}$$

$$\text{Matthews correlation coefficient (MCC)} = \frac{TP\times TN-FP\times FN}{\sqrt{(TP+FP)(TP+FN)(TN+FP)(TN+FN)}}$$

$$\text{Sensitivity} = TPR$$

$$\text{Specificity} = 1-FPR$$

$$\text{Precision} = \frac{TP}{TP+FP}$$

$$\text{Recall} = TPR$$

$$FDR = 1-\text{Precision}$$

Fig. S1 The confusion matrix and definitions of some commonly used measures calculated from it. In these definitions, P, N, TP, FP, FN, and TN are the numbers of entities in these categories based on a specific classification threshold.

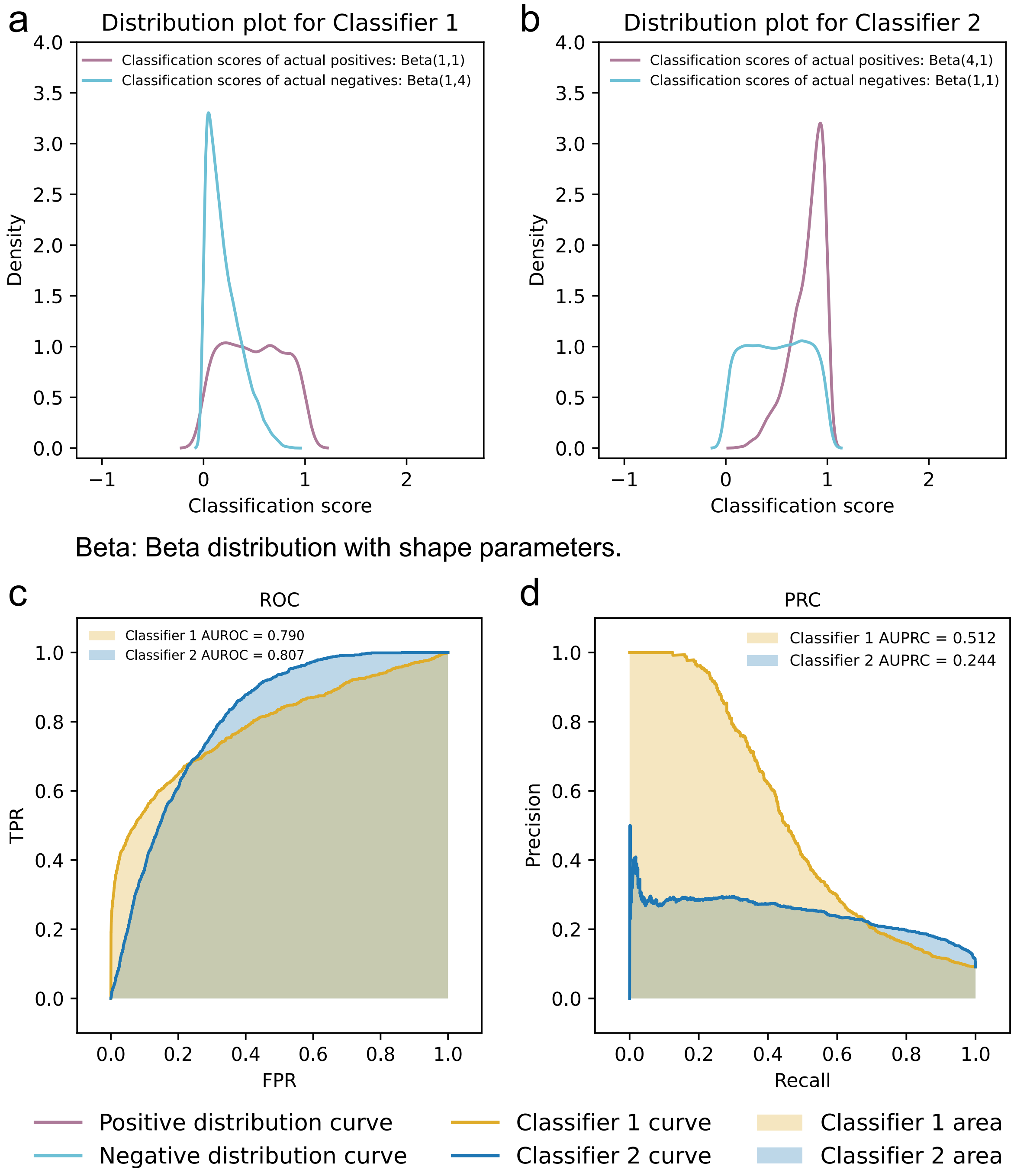

Fig. S2 A demonstration that AUPRC is more sensitive to performance differences than AUROC when the target class has few entities and there are errors among the top predictions. **a,b** Classification scores produced by two classifiers simulated using Beta distributions [1]. A larger proportion of actual positives are predicted among the entities receiving the highest classification scores produced by Classifier 1 than Classifier 2. **c** The ROCs and AUROCs of the two classifiers. Classifier 1 has a slightly smaller AUROC than Classifier 2. **d** The PRCs and AUPRCs of the two classifiers. Classifier 1 has a much larger AUPRC than Classifier 2.

a

Anchor point	Classification score	Actual class	TP	FP	Recall	Precision
A	0.9	0	0	1	0	0
B	0.8	1	1	1	0.5	0.5
C	0.7	0	1	2	0.5	0.333
D	0.6	1	2	2	1	0.5
E	0.5	0	2	3	1	0.4

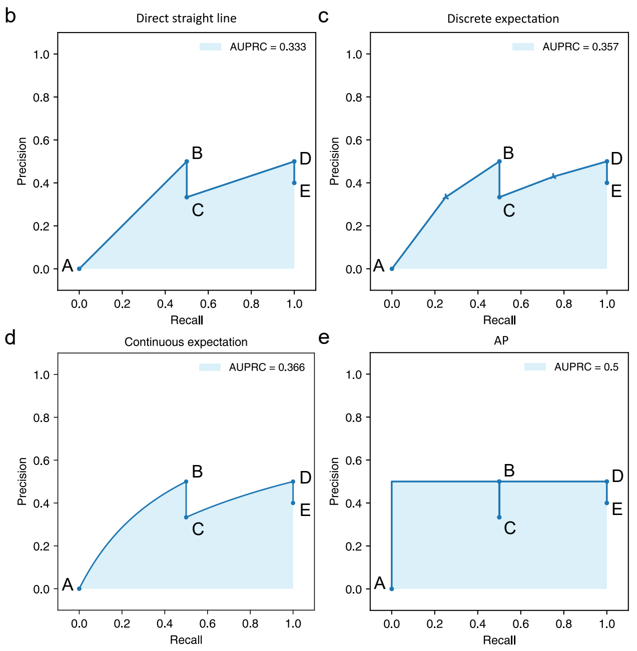

f

Anchor point	Classification score	Actual class	TP	FP	Recall	Precision
A	0.6	0	0	1	0	0
B	0.4	1	2	2	1	0.5
	0.4	1				
	0.4	0				
C	0.3	0	2	3	1	0.4

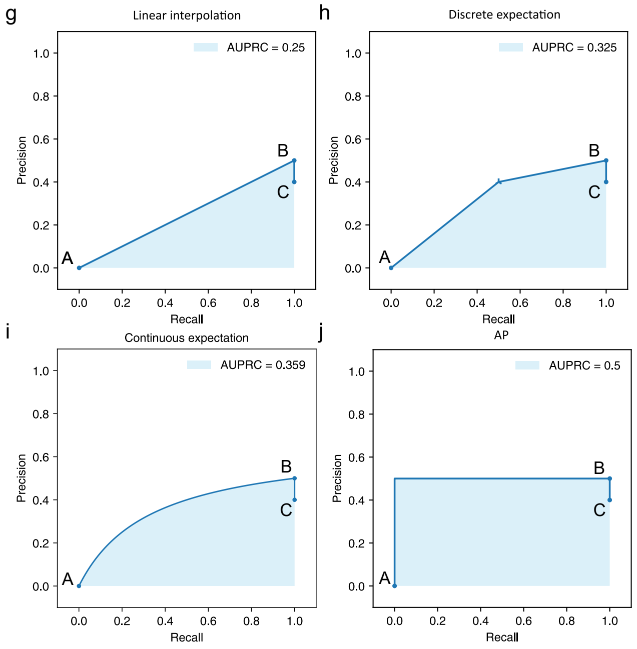

Anchor point — Non-linearly interpolated point — PRC — Area

Fig. S3 Extra examples that illustrative different methods for connecting adjacent anchor points on the PRC. **a** An illustrative data set with no two entities receiving the same classification score. **b-e** Different methods for connecting adjacent anchor points when there are no ties in classification scores, namely **b** direct straight line, **c** discrete expectation, **d** continuous expectation, and **e** AP. **f** An illustrative data set with different entities receiving the same classification score. Each group of entities with the same classification score defines a single anchor point (A, B, and C). **g-j** Different methods for connecting anchor point B to its previous anchor point, A, namely **g** linear interpolation, **h** discrete expectation, **i** continuous expectation, and **j** AP. In **c** and **h**, *tp* is set to 0.5 and 1 in Formula 1, respectively (Additional file 1: Supplementary Text).

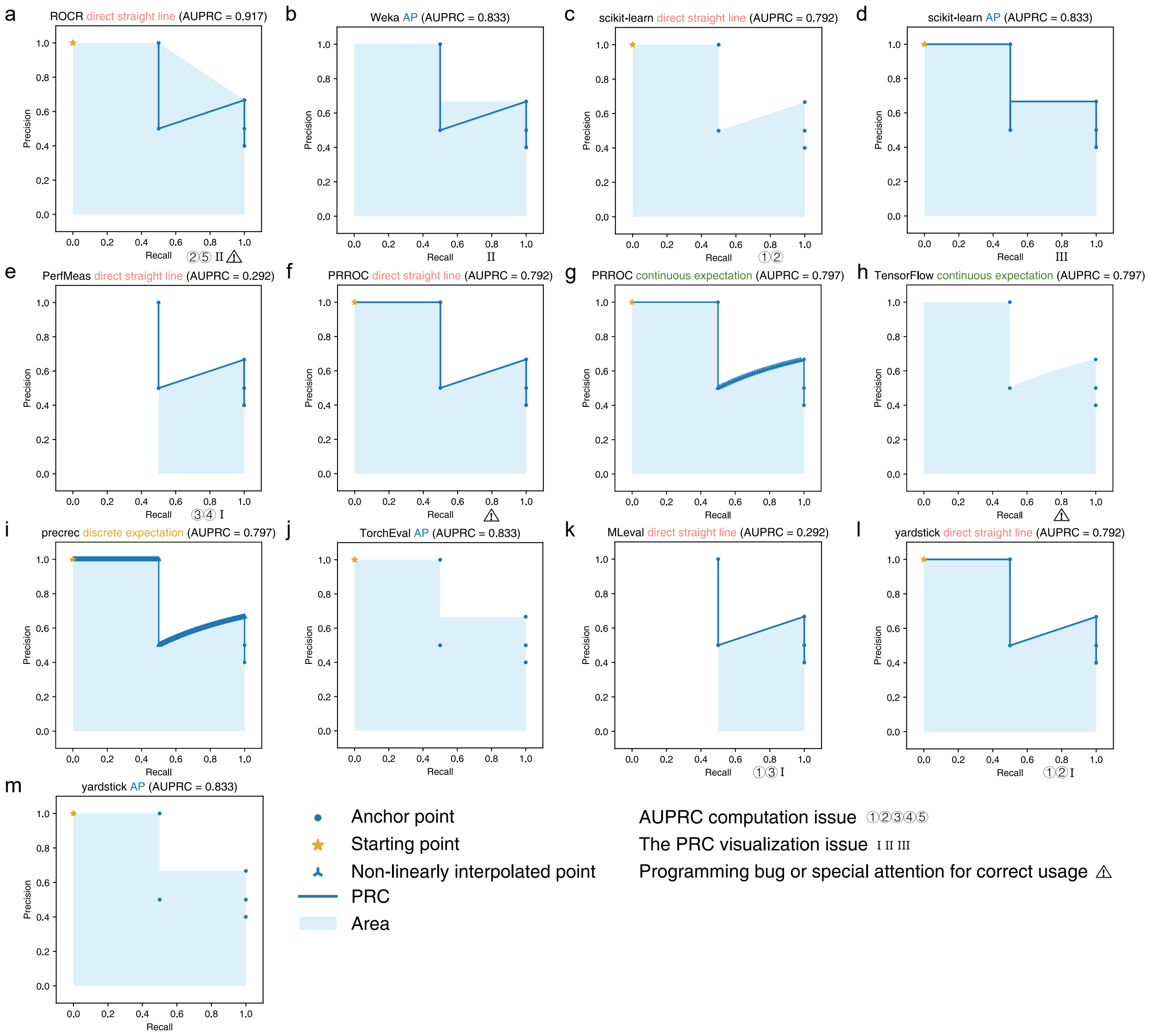

Fig. S4 The PRCs and AUPRCs of the illustrative example in Fig. 1a produced by the 10 tools. **a** ROCR (direct straight line). **b** Weka (AP). **c** scikit-learn (direct straight line). **d** scikit-learn (AP). **e** PerfMeas (direct straight line). **f** PRROC (direct straight line). **g** PRROC (continuous expectation). **h** TensorFlow (continuous expectation). **i** precrec (discrete expectation). **j** TorchEval (AP). **k** MLeval (direct straight line). **l** yardstick (direct straight line). **m** yardstick (AP).

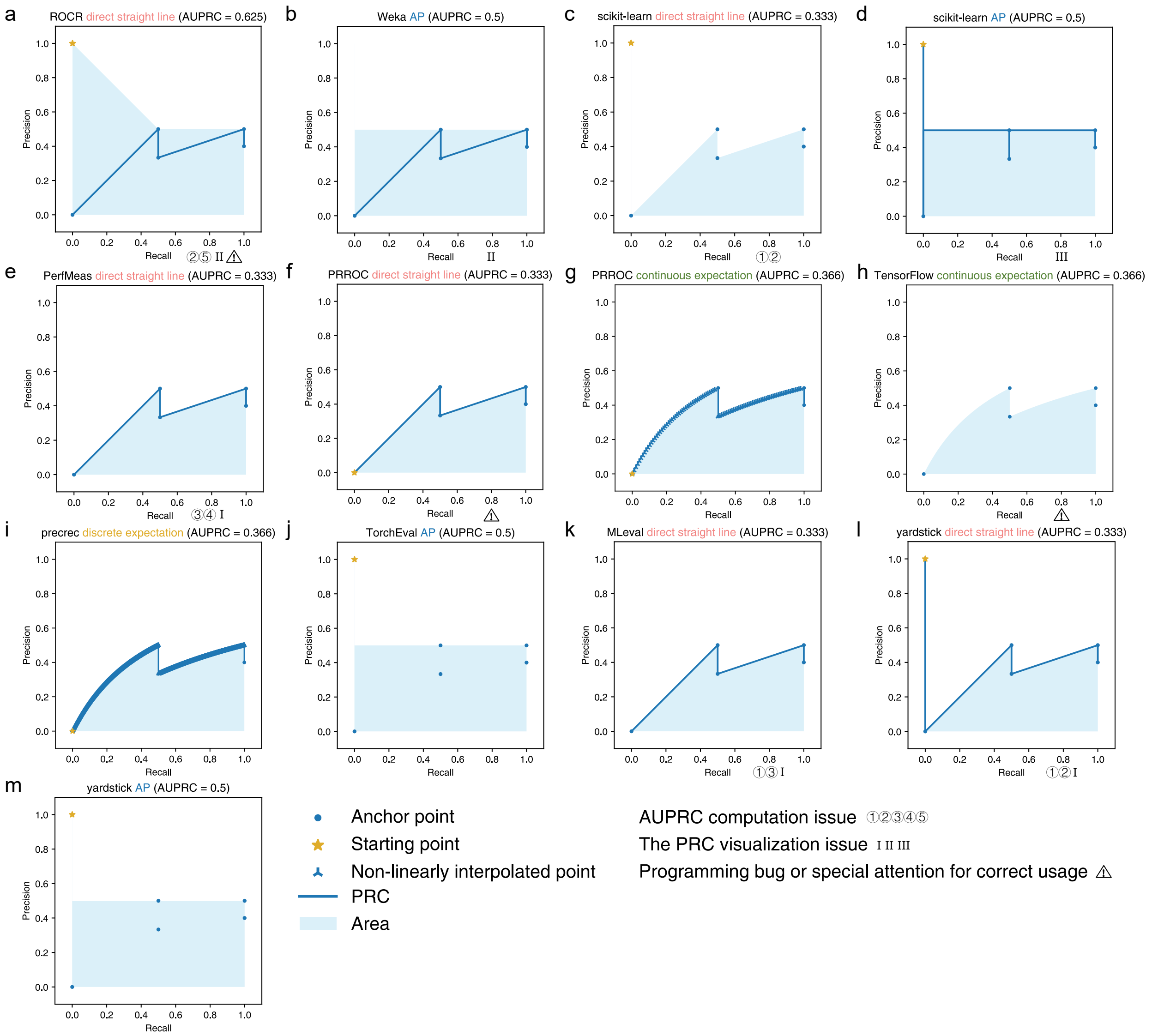

Fig. S5 The PRCs and AUPRCs of the illustrative example in Additional file 1: Fig. S3a produced by the 10 tools. **a** ROCR (direct straight line). **b** Weka (AP). **c** scikit-learn (direct straight line). **d** scikit-learn (AP). **e** PerfMeas (direct straight line). **f** PRROC (direct straight line). **g** PRROC (continuous expectation). **h** TensorFlow (continuous expectation). **i** precrec (discrete expectation). **j** TorchEval (AP). **k** MLeval (direct straight line). **l** yardstick (direct straight line). **m** yardstick (AP).

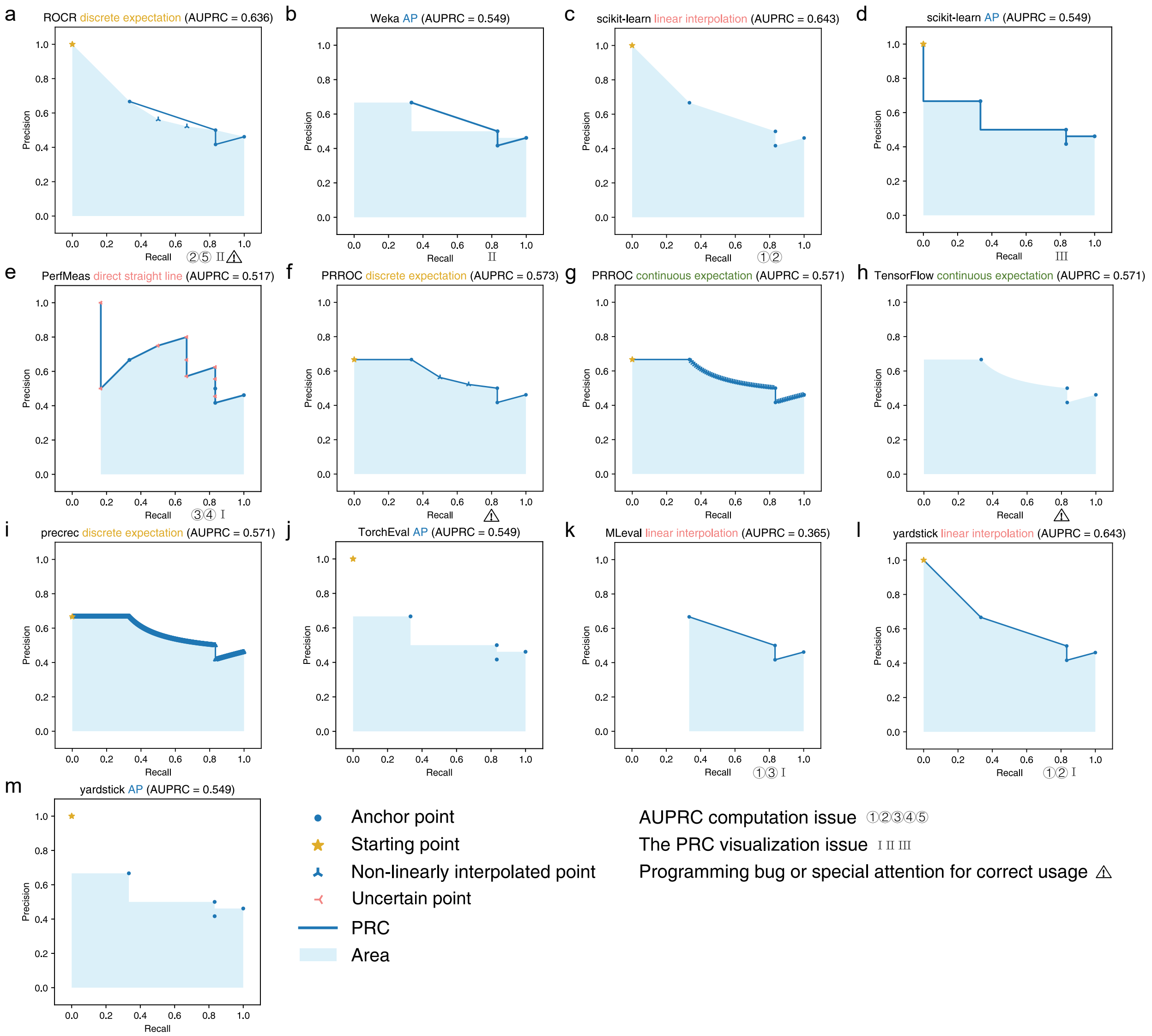

Fig. S6 The PRCs and AUPRCs of the illustrative example in Fig. 1f produced by the 10 tools. **a** ROCR (discrete expectation). **b** Weka (AP). **c** scikit-learn (linear interpolation). **d** scikit-learn (AP). **e** PerfMeas (direct straight line). **f** PRROC (discrete expectation). **g** PRROC (continuous expectation). **h** TensorFlow (continuous expectation). **i** precrec (discrete expectation). **j** TorchEval (AP). **k** MLeval (linear interpolation). **l** yardstick (linear interpolation). **m** yardstick (AP). The uncertain points on the PRC produced by PerfMeas are caused by its dependency on the input order of entities in ties.

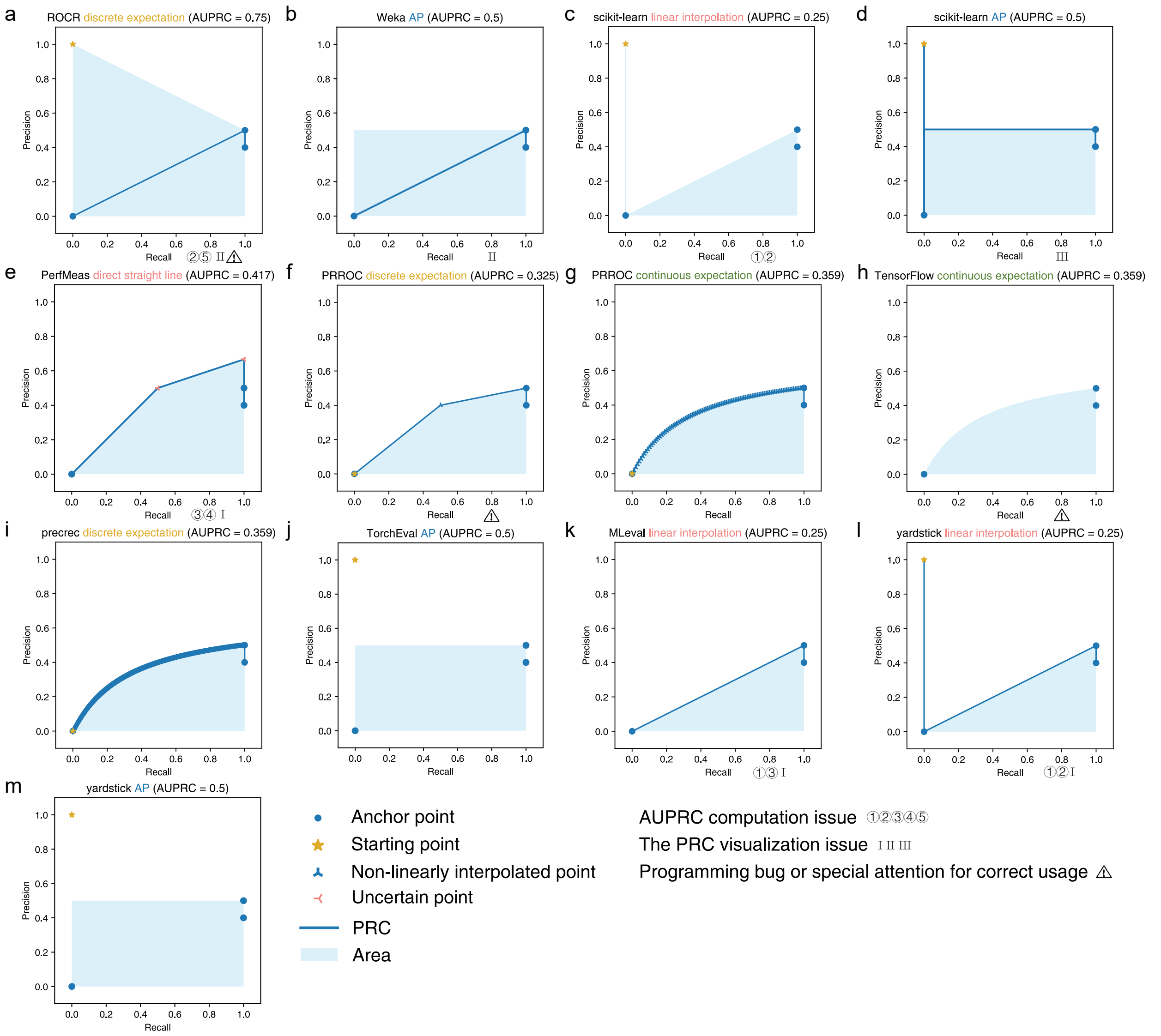

Fig. S7 The PRCs and AUPRCs of the illustrative example in Additional file 1: Fig. S3f produced by the 10 tools. **a** ROCR (discrete expectation). **b** Weka (AP). **c** scikit-learn (linear interpolation). **d** scikit-learn (AP). **e** PerfMeas (direct straight line). **f** PRROC (discrete expectation). **g** PRROC (continuous expectation). **h** TensorFlow (continuous expectation). **i** precrec (discrete expectation). **j** TorchEval (AP). **k** MLeval (linear interpolation). **l** yardstick (linear interpolation). **m** yardstick (AP). The uncertain points on the PRC produced by PerfMeas are caused by its dependency on the input order of entities in ties.

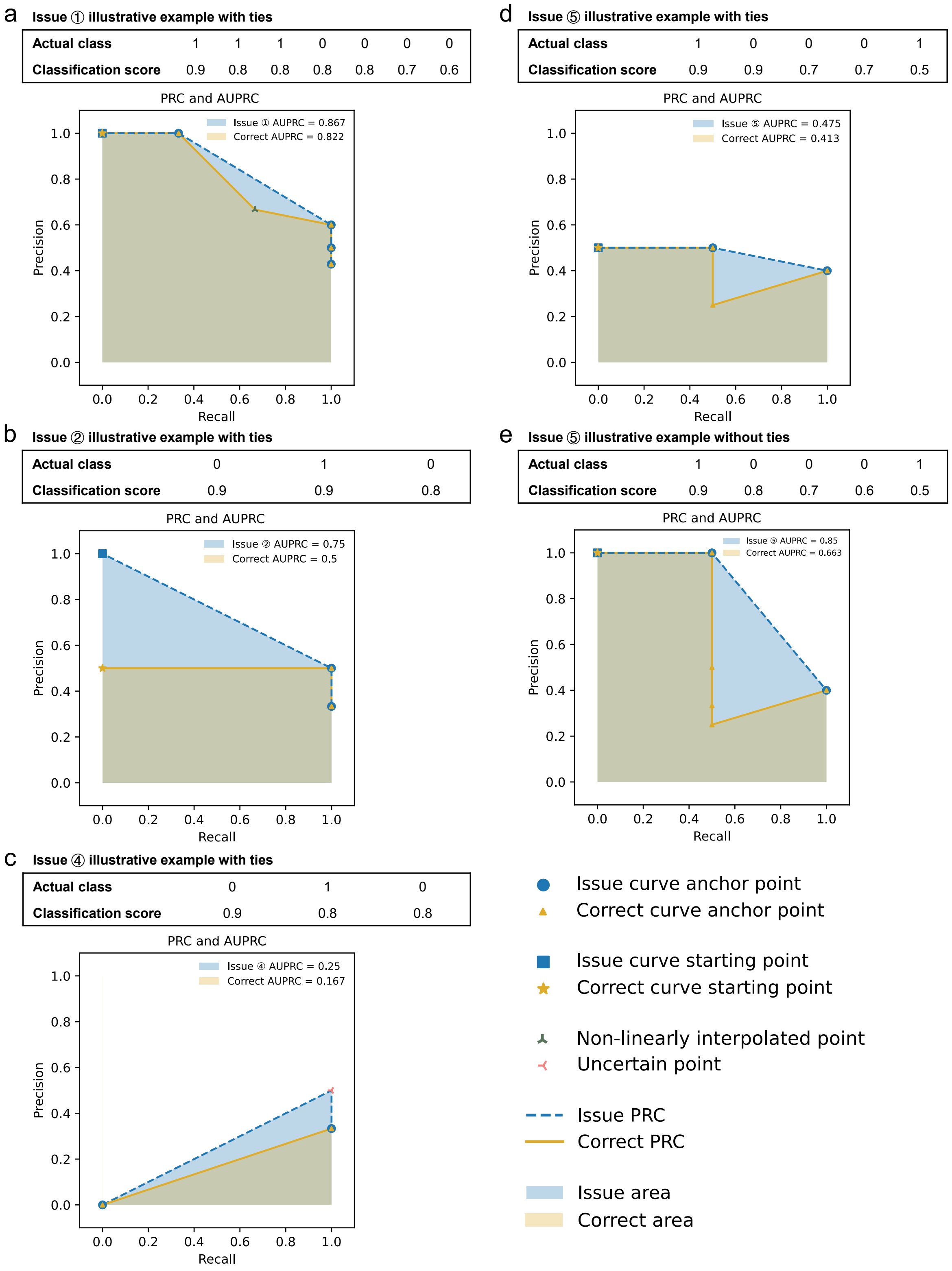

Fig. S8 Illustrative examples of overly optimistic AUPRC values caused by the issues. **a** Issue ①. **b** Issue ②. **c** Issue ④. **d-e** Issue ⑤ with (**d**) or without (**e**) ties in classification scores. The correct PRCs and AUPRCs were generated by the bug-fixed version of PRROC (option that uses the discrete expectation method to handle ties).

a Illustrative example with ties

**Actual class**	1	1	0	0	1	0	0	0
**Classifier 1 classification score**	0.9	0.8	0.8	0.8	0.8	0.8	0.8	0.8
**Classifier 2 classification score**	0.9	0.8	0.8	0.8	0.7	0.7	0.7	0.7

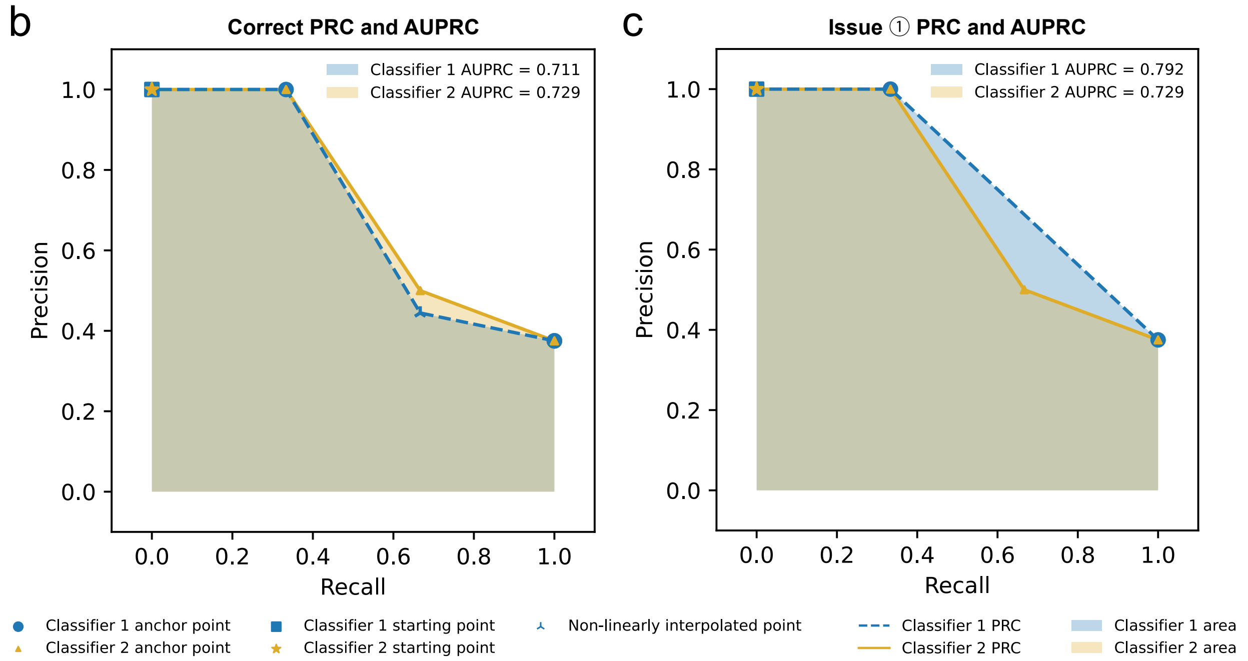

Fig. S9 An illustrative example of flipping the orders of two classifiers based on their AUPRC values due to Issue ① when there are ties in classification scores. **a** An illustrative data set. **b** The PRCs and AUPRCs of two classifiers produced by the bug-fixed version of PRROC (option that uses the discrete expectation method to handle ties). **c** The PRCs and AUPRCs of the two classifiers with Issue ①.

a Illustrative example with ties

Actual class	0	1	0	0	1	0	0	1
**Classifier 1 classification score**	0.7	0.7	0.7	0.7	0.7	0.6	0.6	0.6
**Classifier 2 classification score**	0.8	0.8	0.7	0.7	0.7	0.6	0.6	0.6

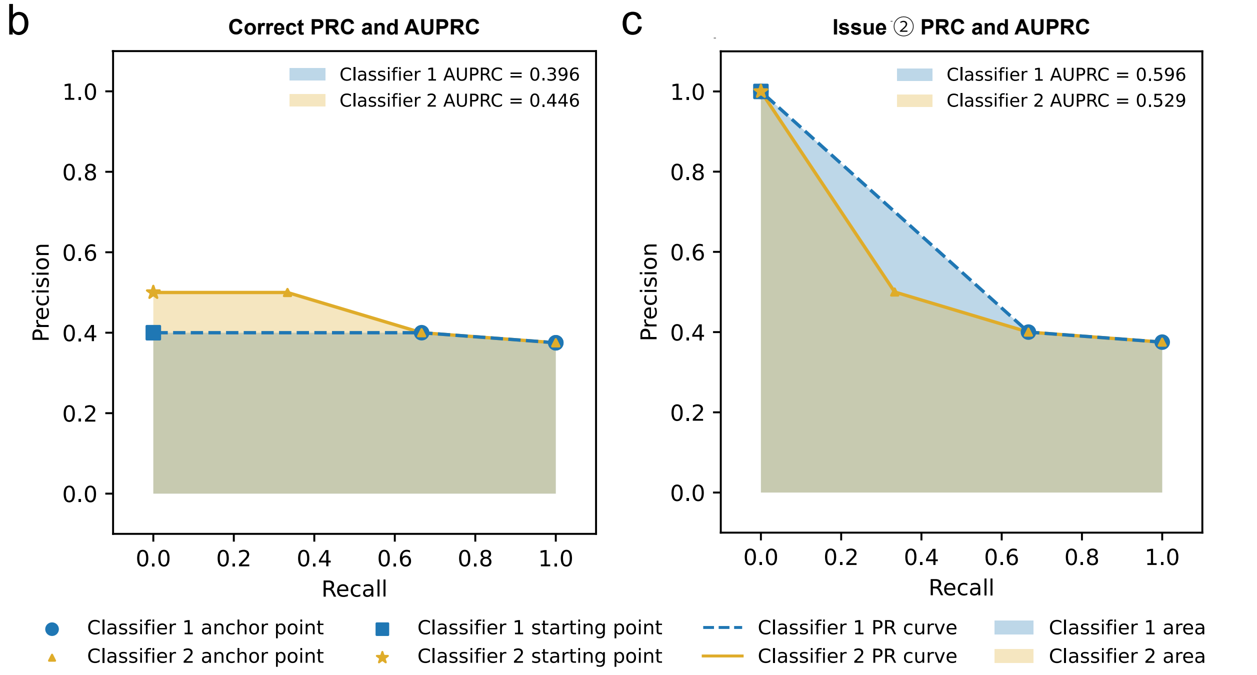

Fig. S10 An illustrative example of flipping the orders of two classifiers based on their AUPRC values due to Issue ② when there are ties in classification scores. **a** An illustrative data set. **b** The PRCs and AUPRCs of two classifiers produced by the bug-fixed version of PRROC (option that uses the discrete expectation method to handle ties). **c** The PRCs and AUPRCs of the two classifiers with Issue ②.

a **Illustrative example with ties**

**Actual class**	1	0	1	0	0	1	0
**Classifier 1 classification score**	0.6	0.6	0.6	0.5	0.5	0.5	0.5
**Classifier 2 classification score**	0.6	0.6	0.5	0.5	0.4	0.4	0.4

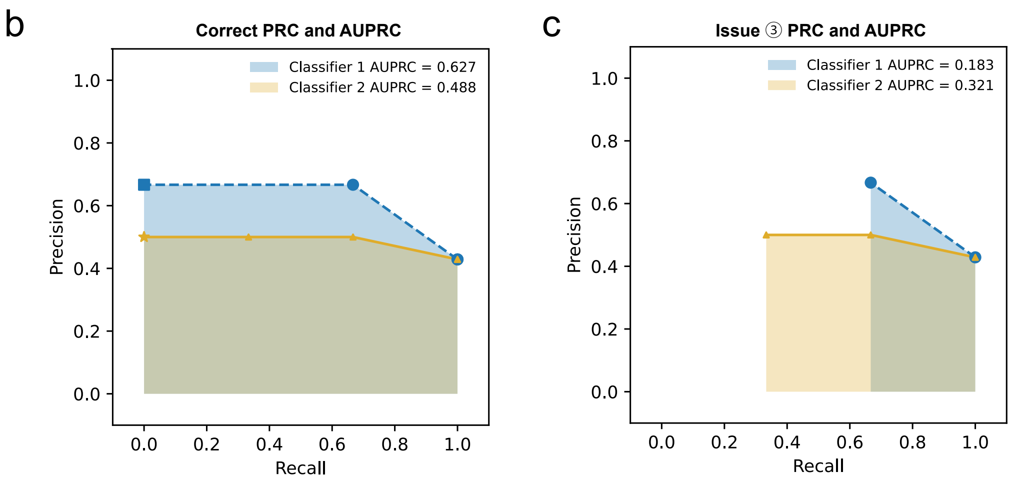

d **Illustrative example without ties**

**Actual class**	1	0	1	1	0	1	0	0	0
**Classifier 3 classification score**	0.9	0.8	0.7	0.6	0.5	0.4	0.3	0.2	0.1
**Classifier 4 classification score**	0.8	0.9	0.7	0.6	0.5	0.4	0.3	0.2	0.1

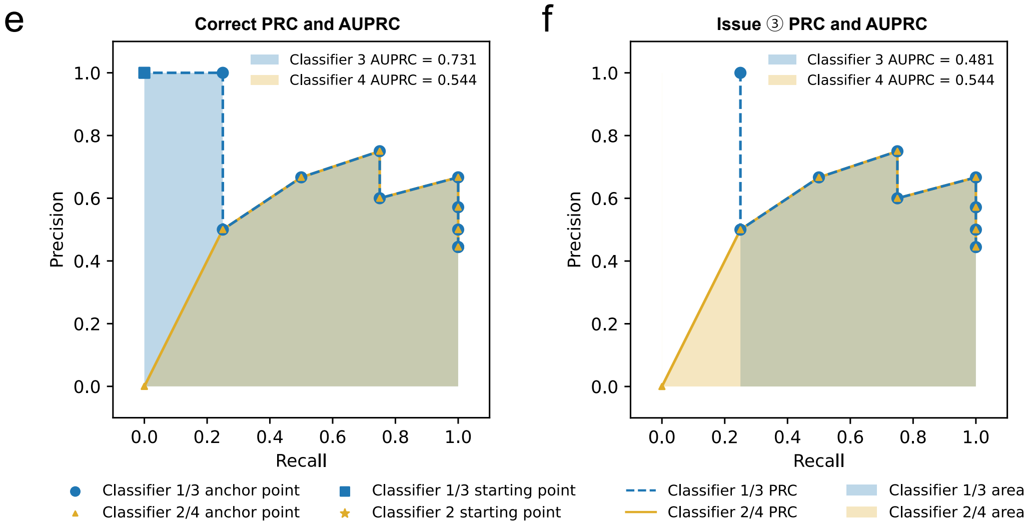

Fig. S11 Illustrative examples of flipping the orders of two classifiers based on their AUPRC values due to Issue ③ with and without ties in classification scores. **a,d** Illustrative data sets with (**a**) and without (**d**) ties. **b,e** The PRCs and AUPRCs of two classifiers produced by the bug-fixed version of PRROC (option that uses the discrete expectation method to handle ties) on the illustrative data set with (**b**) and without (**e**) ties. **c,f** The PRCs and AUPRCs of the two classifiers with Issue ③ based on the illustrative data set with (**c**) and without (**f**) ties.

a **Illustrative example with ties (the same classifier but different output orders)**

Classifier 1 classification score	0.8	0.7	0.6	0.6	0.5	0.3	0.2
Classifier 1 actual class order 1	1	0	0	1	1	0	0
Classifier 1 actual class order 2	1	0	1	0	1	0	0

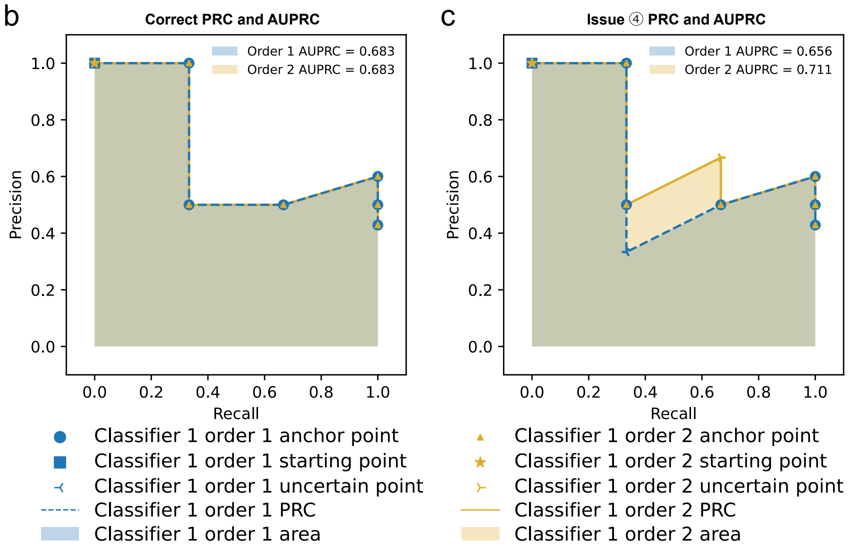

d **Illustrative example with ties (different classifiers)**

Actual class	0	1	0	0	1
Classifier 2 classification score	0.8	0.7	0.6	0.6	0.6
Classifier 3 classification score	0.8	0.7	0.6	0.4	0.5

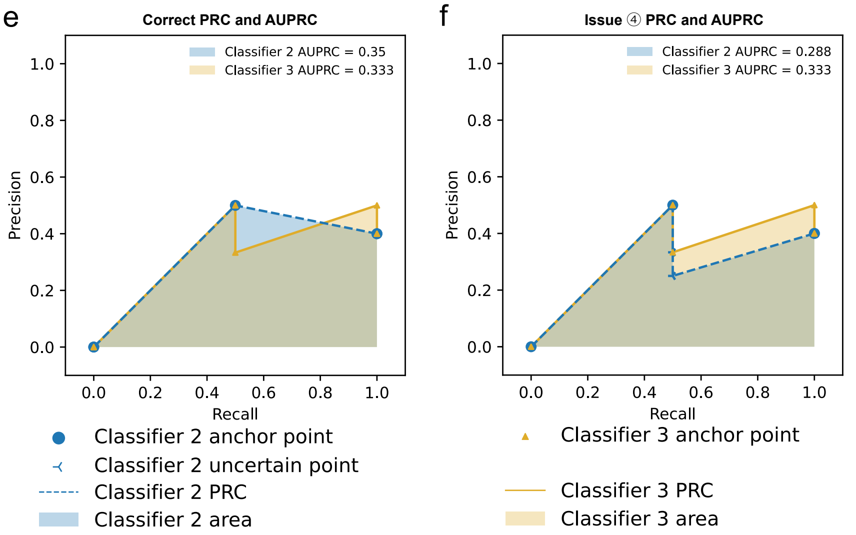

Fig. S12 Illustrative examples of Issue ④. **a** An illustrative data set with ties in classification scores produced by a classifier and two different input orders of the entities. **b** The correct PRC and AUPRC, which stays the same regardless of the input order of the entities. **c** The PRCs and AUPRCs produced if entities with the same classification score are ordered based on their input order. **d** An illustrative data set with ties in classification scores produced by one of the two classifiers. **e,f** The PRCs and AUPRCs of the two classifiers with Issue ④.

a Illustrative example with ties

**Actual class**	1	0	0	1	0
**Classifier 1 classification score**	0.9	0.7	0.7	0.6	0.5
**Classifier 2 classification score**	0.9	0.7	0.7	0.7	0.7

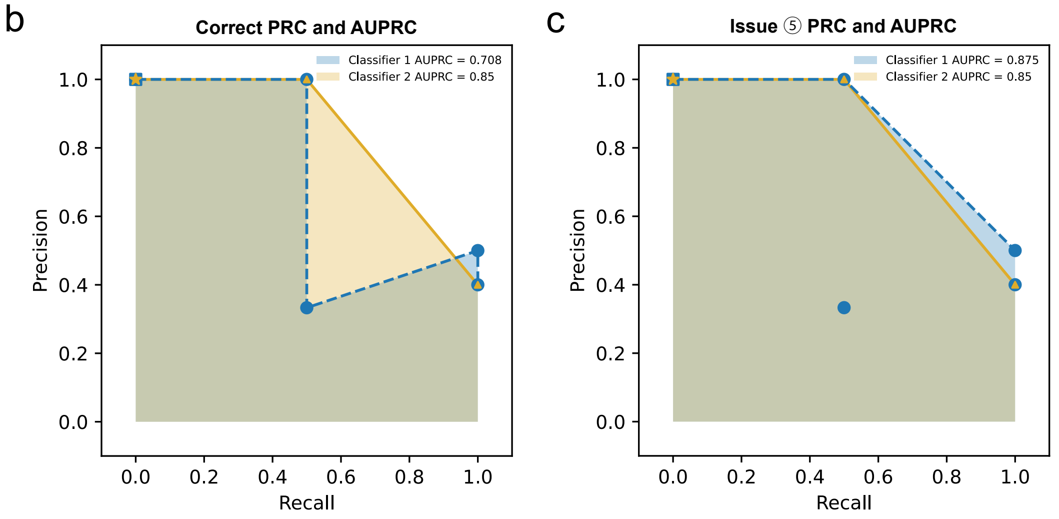

d Illustrative example without ties

**Actual class**	1	0	1	0	0	0	1
**Classifier 3 classification score**	0.9	0.8	0.7	0.6	0.5	0.4	0.3
**Classifier 4 classification score**	0.9	0.8	0.6	0.7	0.4	0.3	0.5

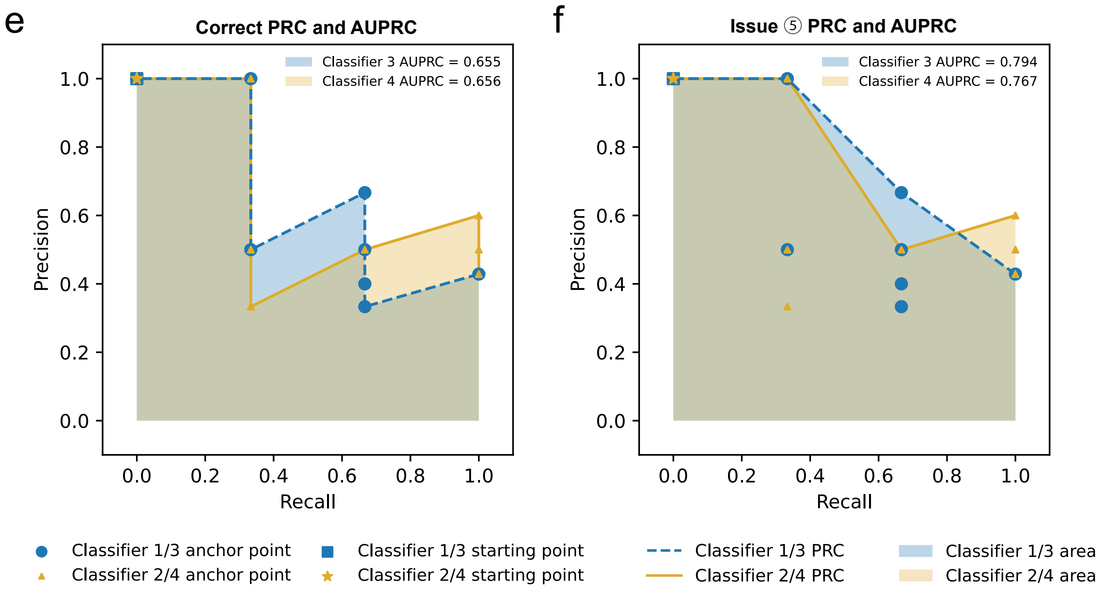

Fig. S13 Illustrative examples of flipping the order of two classifiers based on their AUPRC values due to Issue ⑤ with and without ties in classification scores. **a,d** Illustrative data sets with (**a**) and without (**d**) ties. **b,e** The PRCs and AUPRCs of two classifiers produced by the bug-fixed version of PRROC (option that uses the discrete expectation method to handle ties). **c,f** The PRCs and AUPRCs of the two classifiers with Issue ⑤ on the illustrative data set with (**c**) and without (**f**) ties.

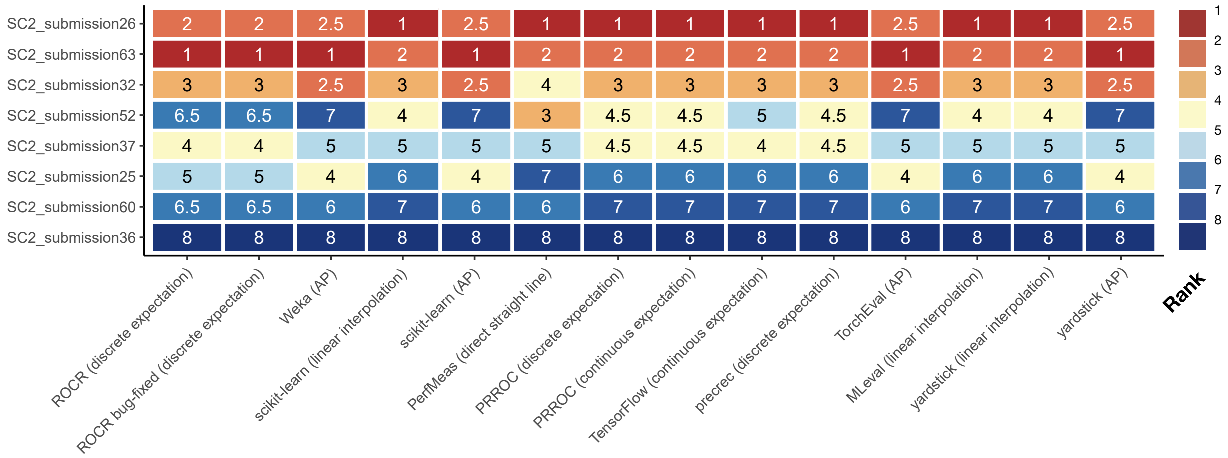

Fig. S14 Ranks of the top 8 submissions to the sbv IMPROVER challenge based on the AUPRC values produced by the 10 tools. Each entry shows the rank of a submission based on the corresponding AUPRC value shown in Fig. 2b.

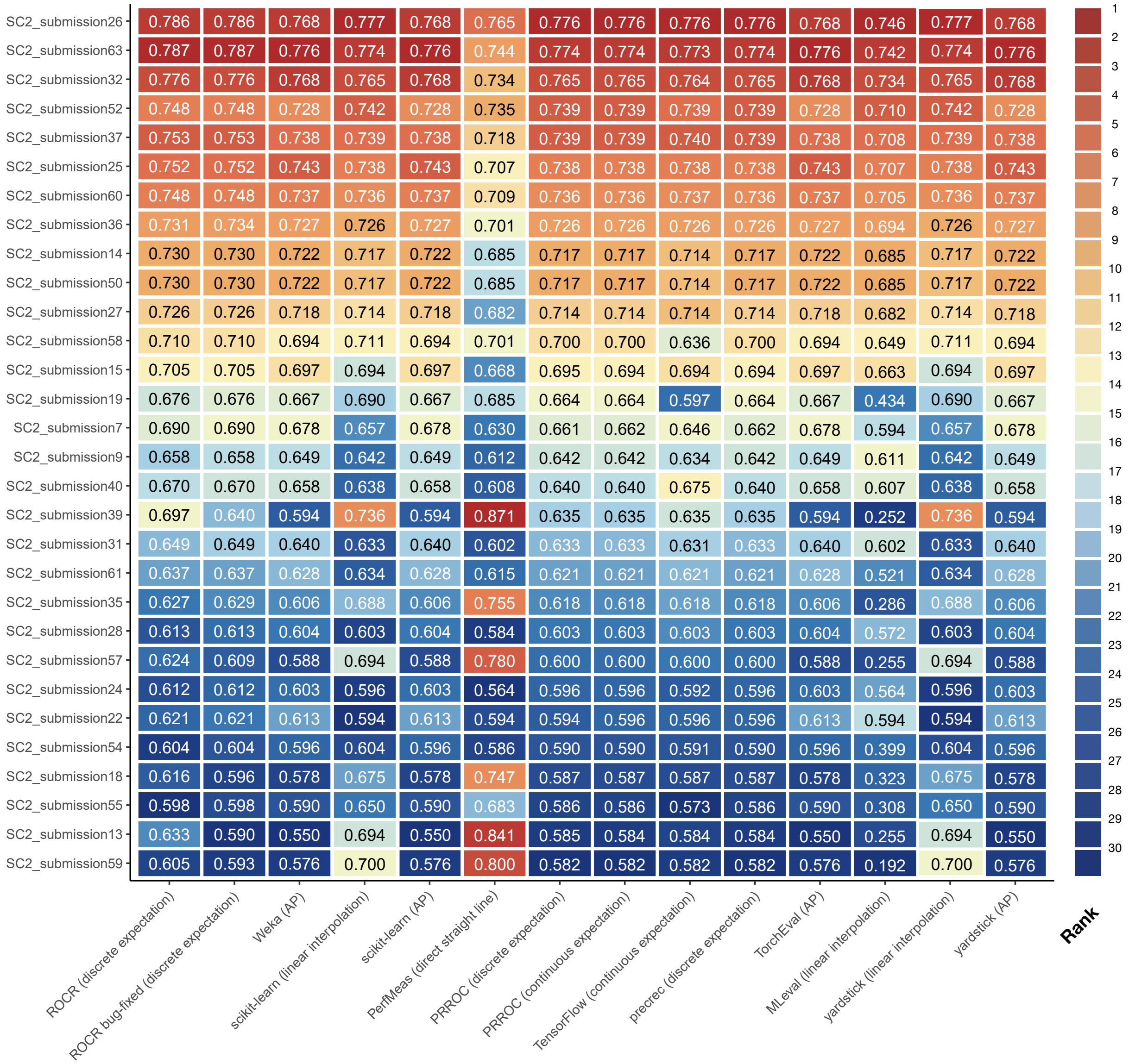

Fig. S15 Ranks of the top 30 submissions to the sbv IMPROVER challenge based on the AUPRC values produced by the 10 tools. Each entry shows the AUPRC value and the background color indicates its rank among the submissions.

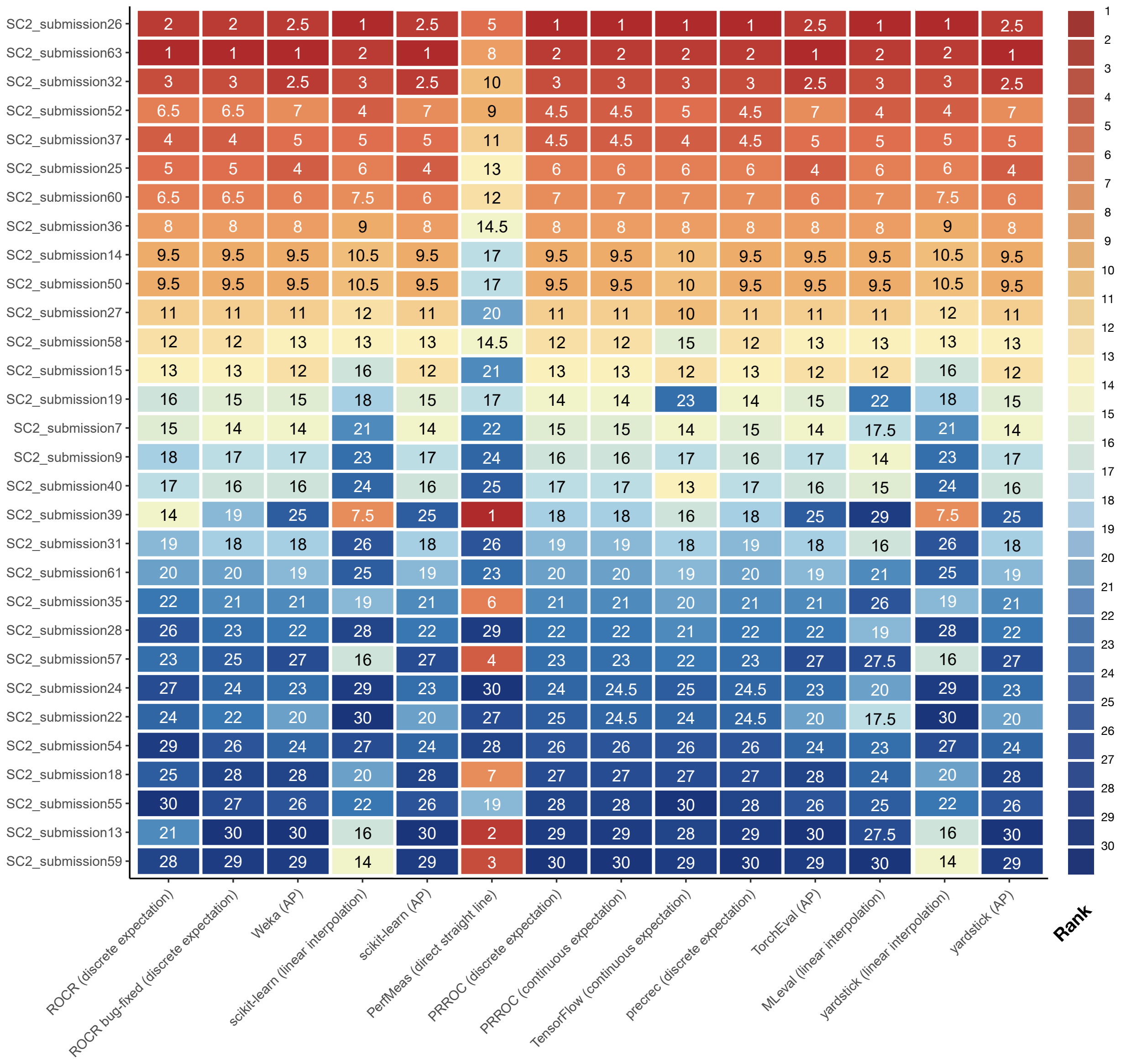

Fig. S16 Ranks of the top 30 submissions to the sbv IMPROVER challenge based on the AUPRC values produced by the 10 tools. Each entry shows the rank of a submission based on the corresponding AUPRC value shown in Additional file 1: Fig. S15.

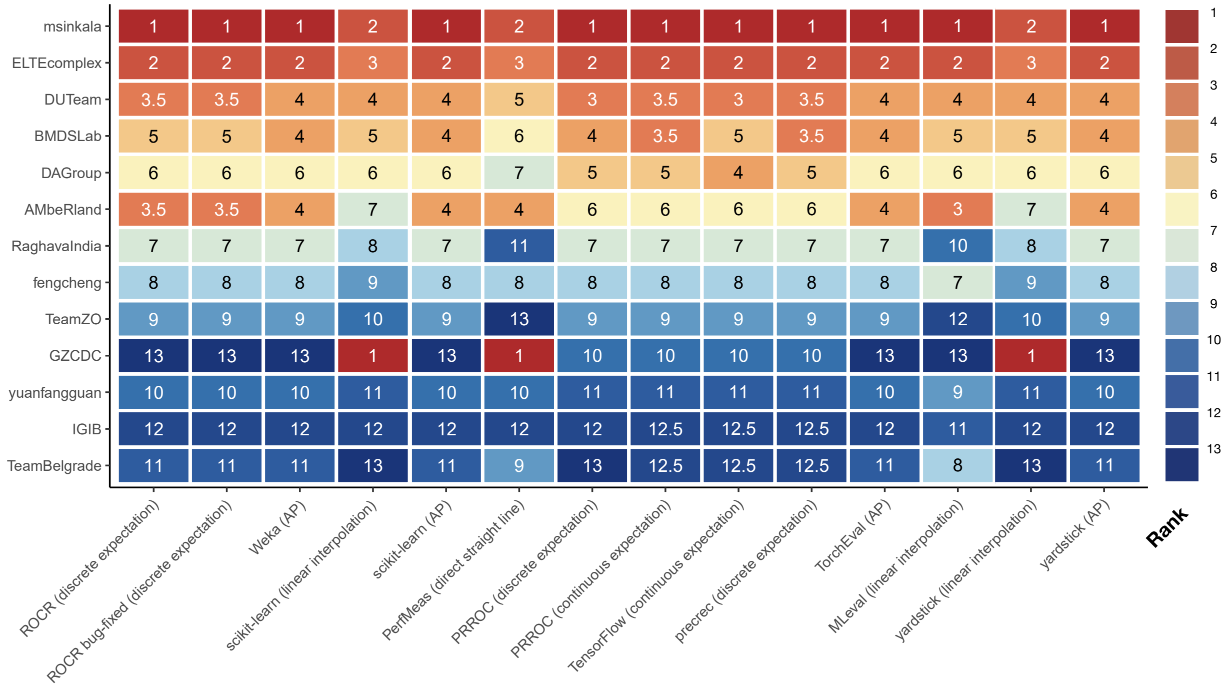

Fig. S17 Ranks of the 13 participants of the DREAM Preterm Birth Prediction Challenge based on the AUPRC values produced by the 10 tools. Each entry shows the rank of a submission based on the corresponding AUPRC value shown in Fig. 2c.

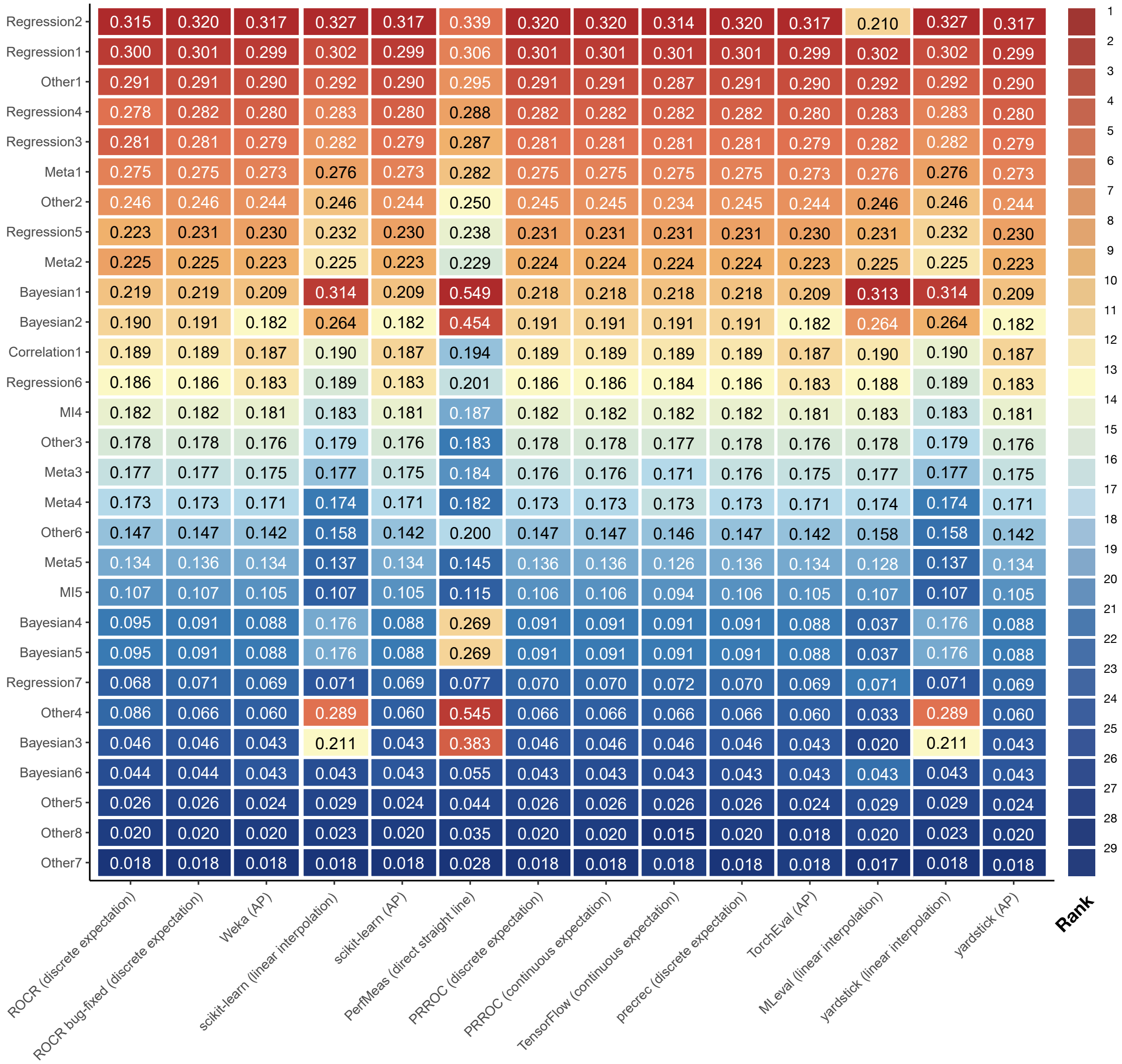

Fig. S18 Ranks of the 29 submissions to the DREAM5 transcription factor target challenge based on the AUPRC values produced by the 10 tools. Each entry shows the AUPRC value and the background color indicates its rank among the submissions. Submission "Other8" obtained an AUPRC value of 0.018 from TorchEval (AP), but 0.020 from the other AP method-based tools. This difference was due to TorchEval's requirement for having the classification scores provided in the form of Tensor objects as input to PyTorch, which led to loss of numeric precision.

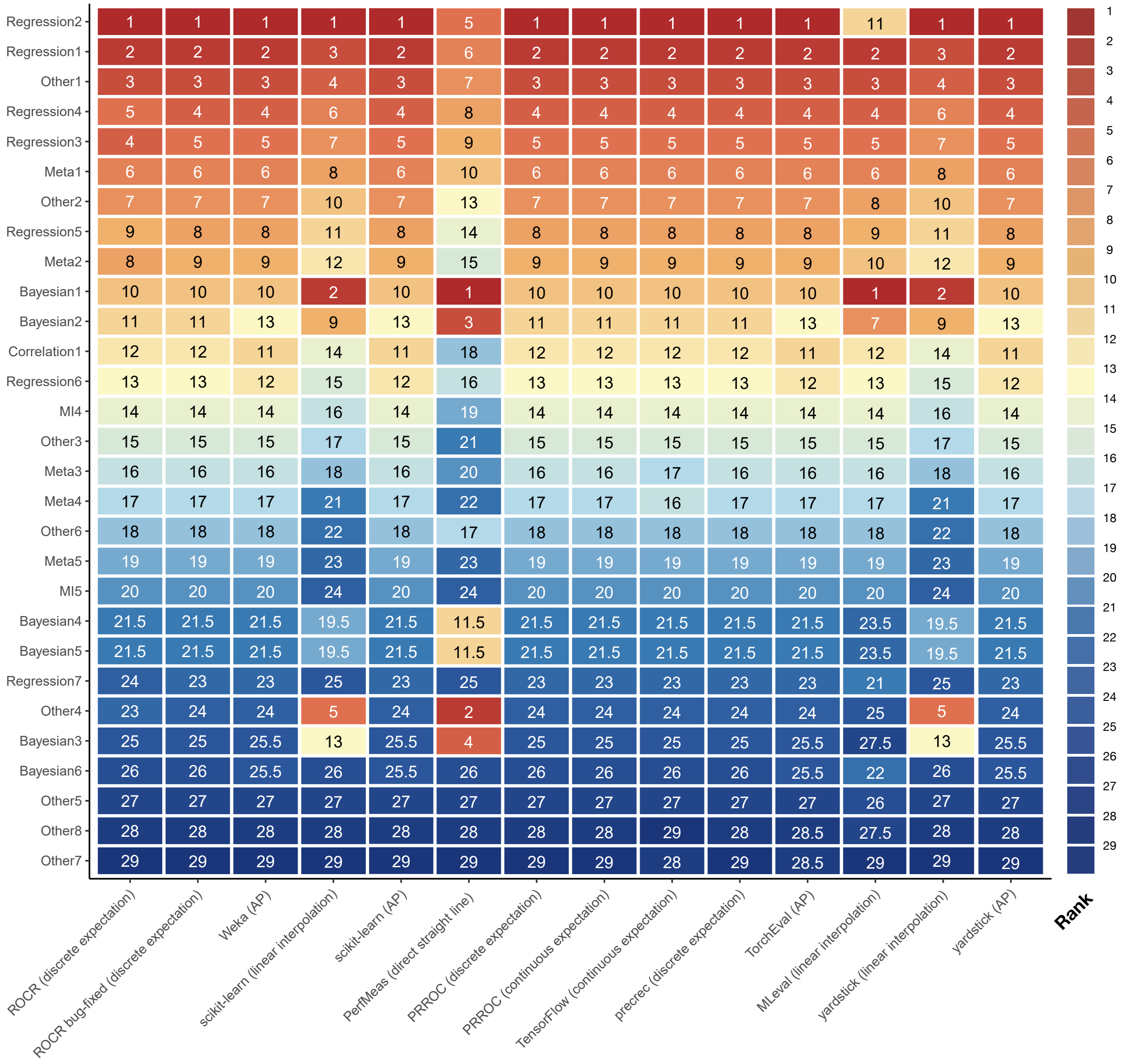

Fig. S19 Ranks of the 29 submissions to the DREAM5 transcription factor target challenge based on the AUPRC values produced by the 10 tools. Each entry shows the rank of a submission based on the corresponding AUPRC value shown in Additional file 1: Fig. S18.

a

Actual class	1	0	1	1	0	0	0	1	1	0	1	1	0	1	1	0	0	0	0	1
**Classification score**	0.7	0.7	0.7	0.6	0.6	0.5	0.5	0.5	0.5	0.5	0.5	0.5	0.5	0.5	0.5	0.5	0.5	0.4	0.3	0.2

b

Incorrect order and incorrect PRC generated by PRROC

Recall	Precision	Threshold
1	0.5	0.2
1	0.5	0.2
0.9	0.529	0.5
0.9	0.474	0.3
0.9	0.5	0.4
0.9	0.529	0.5
0.8	0.533	0.5
0.7	0.538	0.5
0.6	0.545	0.5
0.5	0.556	0.5
0.4	0.571	0.5
0.3	0.6	0.5
0.3	0.6	0.6
0.2	0.667	0.7
0	0.667	0.7

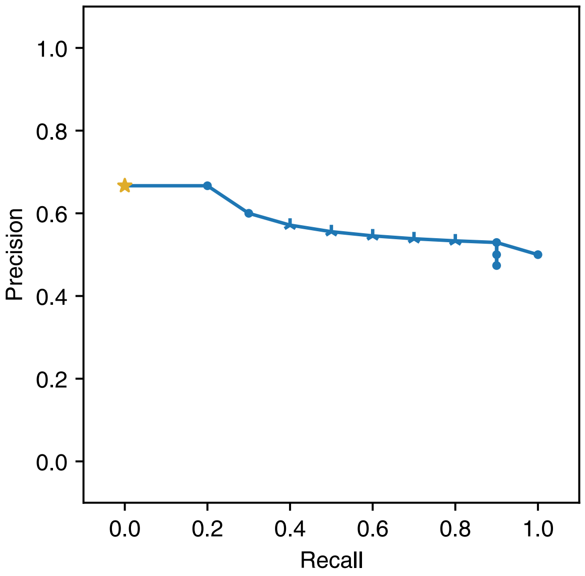

c

Correct order and correct PRC

Recall	Precision	Threshold
1	0.5	0.2
1	0.5	0.2
0.9	0.474	0.3
0.9	0.5	0.4
0.9	0.529	0.5
0.9	0.529	0.5
0.8	0.533	0.5
0.7	0.538	0.5
0.6	0.545	0.5
0.5	0.556	0.5
0.4	0.571	0.5
0.3	0.6	0.5
0.3	0.6	0.6
0.2	0.667	0.7
0	0.667	0.7

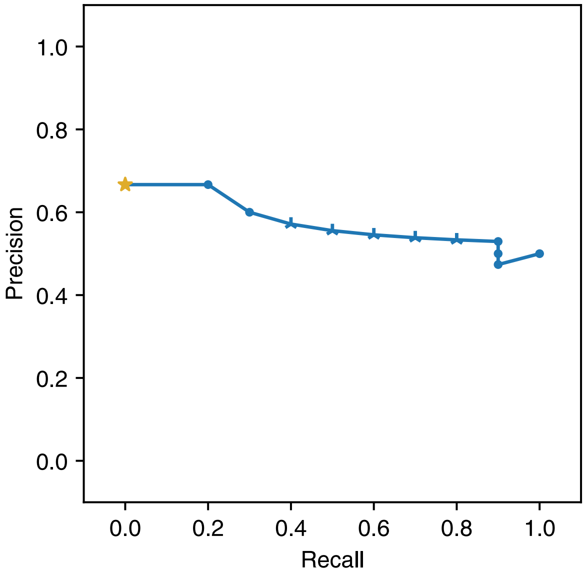

Anchor point   Starting point   Non-linearly interpolated point   PRC

Fig. S20 Programming bug of PRROC (option that uses the discrete expectation method to handle ties) when visualizing the PRC. **a** An illustrative data set. **b** Incorrectly ordered thresholds (highlighted in red) produced by the original version of PRROC and the resulting PRC. **c** The correctly ordered threshold and the resulting PRC produced by the bug-fixed version of PRROC.

a

```
# This example is excerpted from TensorFlow's official website:
https://tensorflow.google.cn/api_docs/python/tf/keras/metrics/AUC
m = tf.keras.metrics.AUC(num_thresholds=3)
m.update_state([0, 0, 1, 1], [0, 0.5, 0.3, 0.9])
# threshold values are [0 - 1e-7, 0.5, 1 + 1e-7]
# tp = [2, 1, 0], fp = [2, 0, 0], fn = [0, 1, 2], tn = [0, 2, 2]
# tp_rate = recall = [1, 0.5, 0], fp_rate = [1, 0, 0]
# auc = ((((1+0.5)/2)*(1-0)) + (((0.5+0)/2)*(0-0))) = 0.75
m.result().numpy()
```

b

No. of thresholds	Thresholds	AUROC (Correct AUROC = 0.75)	AUPRC (Correct AUPRC = 0.797)	TP	FP
2	-1e-7, 1+1e-7	0.5	0.5	2,0	2,0
3 (by the official example)	-1e-7, 0.5, 1+1e-7	0.75	0.821	2,1,0	2,0,0
4	-1e-7, 0.333, 0.667, 1+1e-7	0.625	0.75	2,1,1,0	2,1,0,0
5	-1e-7, 0.25, 0.5, 0.75, 1+1e-7	0.875	0.887	2,2,1,1,0	2,1,0,0,0
6	-1e-7, 0.2, 0.4, 0.6, 0.8, 1+1e-7	0.75	0.797	2,2,1,1,1,0	2,1,1,0,0,0
7	-1e-7, 0.167, 0.333, 0.5, 0.667, 0.833, 1+1e-7	0.75	0.797	2,2,1,1,1,1,0	2,1,1,0,0,0,0
8	-1e-7, 0.143, 0.286, 0.429, 0.571, 0.714, 0.857, 1+1e-7	0.75	0.797	2,2,2,1,1,1,1,0	2,1,1,1,0,0,0,0
9	-1e-7, 0.125, 0.25, 0.375, 0.5, 0.625, 0.75, 0.875, 1+1e-7	0.75	0.797	2,2,2,1,1,1,1,1,0	2,1,1,1,0,0,0,0,0
10	-1e-7, 0.111, 0.222, 0.333, 0.444, 0.556, 0.667, 0.778, 0.889, 1+1e-7	0.75	0.797	2,2,2,1,1,1,1,1,1,0	2,1,1,1,1,0,0,0,0,0

Fig. S21 The user-defined number of thresholds can affect TensorFlow's AUPRC and AUROC calcualtions.**a** An example provided on TensorFlow's official web site, which uses 3 thresholds. **b** The AUPRC and AUROC values computed by TensorFlow with different numbers of thresholds. The "correct" values, as computed by PRROC using the continuous expectation method to handle ties, are shown in the column headers.

a

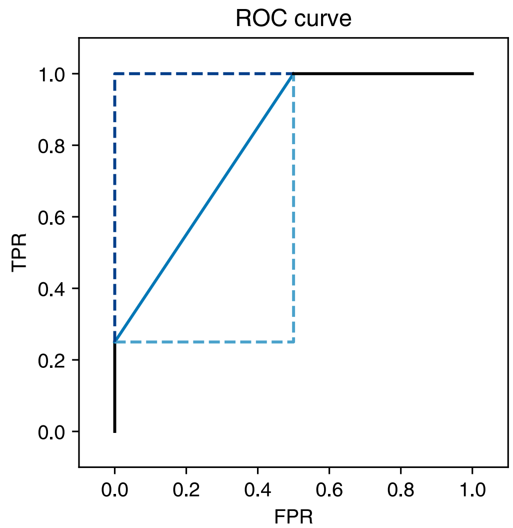

b

Classification Score	Actual Class	TP	FP	TPR	FPR
0.9	1	1	0	0.25	0
0.8	1				
0.8	1				
0.8	1	4	2	1	0.5
0.8	0				
0.8	0				
0.7	0	4	4	1	1
0.7	0				

Expected ROC (AUROC = 0.813)

c

Classification Score	Actual Class	TP	FP	TPR	FPR
0.9	1	1	0	0.25	0
0.8	1	2	0	0.5	0
0.8	1	3	0	0.75	0
0.8	1	4	0	1	0
0.8	0	4	1	1	0.25
0.8	0	4	2	1	0.5
0.7	0	4	4	1	1
0.7	0				

Most optimistic ROC (AUROC = 1)

d

Classification Score	Actual Class	TP	FP	TPR	FPR
0.9	1	1	0	0.25	0
0.8	0	1	1	0.25	0.25
0.8	0	1	2	0.25	0.5
0.8	1	2	2	0.5	0.5
0.8	1	3	2	0.75	0.5
0.8	1	4	2	1	0.5
0.7	0	4	4	1	1
0.7	0				

Most pessimistic ROC (AUROC = 0.625)

Fig. S22 The expected, most optimistic, and most pessimistic ROC curves based on different ways to handle ties in classification scores. **a** The expected ROC curve (the black solid lines and the blue solid line), the most optimistic ROC curve (the black solid lines and the dark blue dashed lines), and most pessimistic ROC curve (the black solid lines and the light blue dashed lines). **b-d** How ties are handled in the three cases, namely taking the average of all possible orders in the expected case (**b**), ordering all actually positive entities first in the most optimistic case (**c**), and ordering all actually negative entities first in the most pessimistic case (**c**).

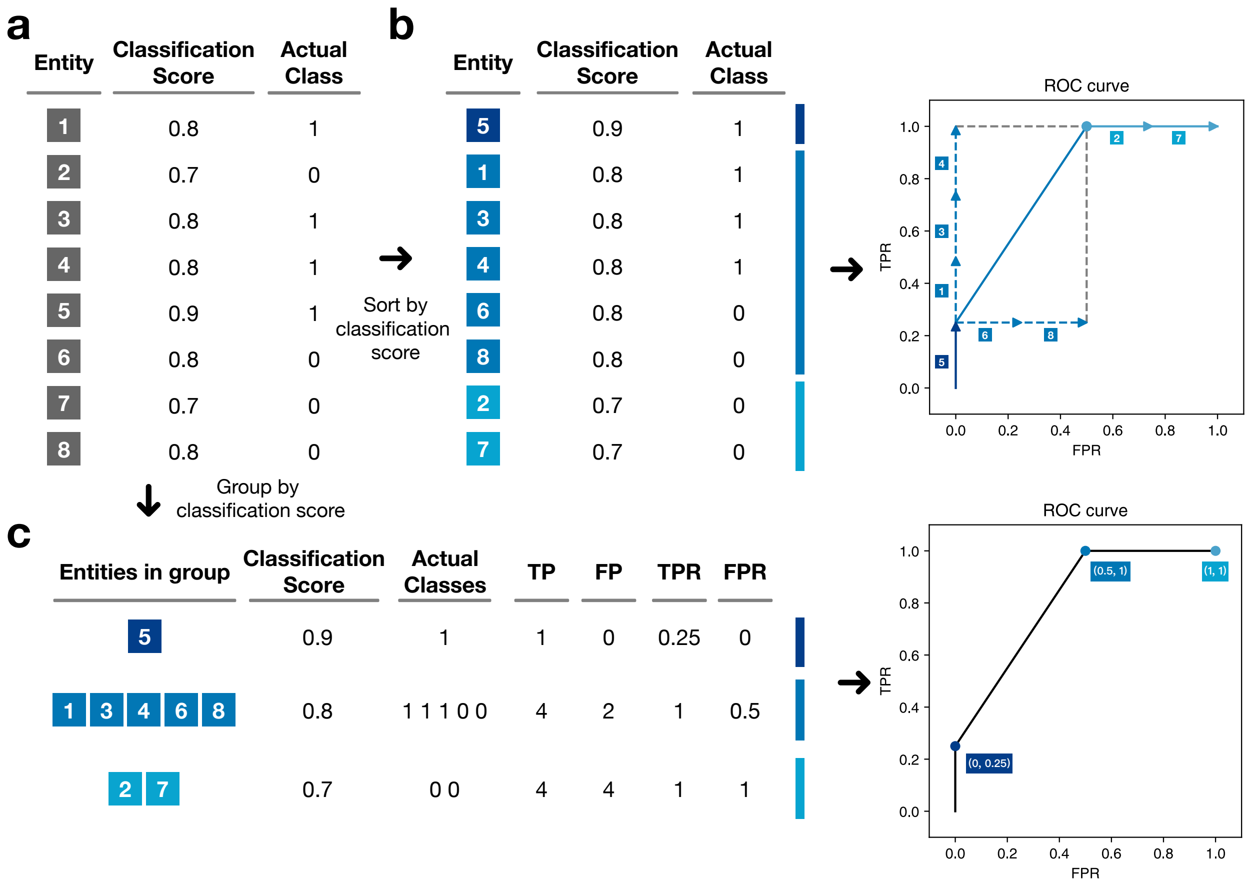

Fig. S23 Two algorithms for constructing the ROC curve. **a** An example data set. **b** The workflow of the first algorithm. **c** The workflow of the second algorithm.

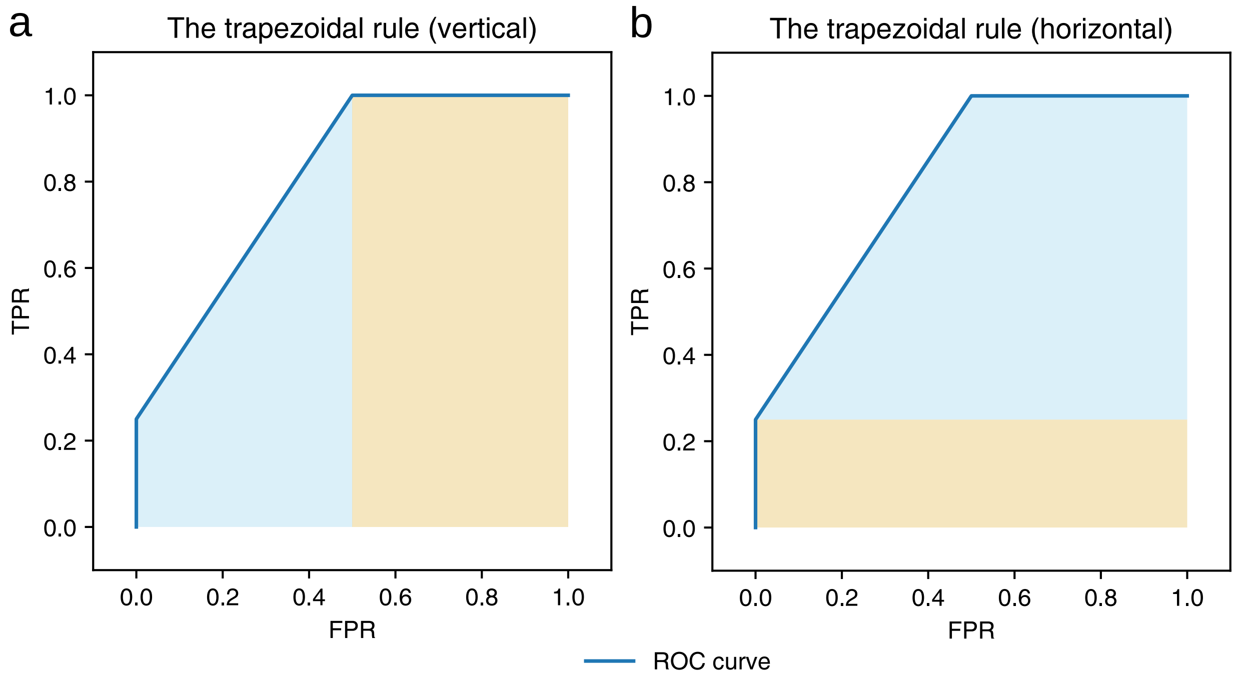

Fig. S24 Computing AUROC by the trapezoidal algorithm. The area is divided either vertically (**a**) or horizontally (**b**) before applying the trapezoidal rule. In this example, AUROC is computed by adding up the blue area and the yellow area.

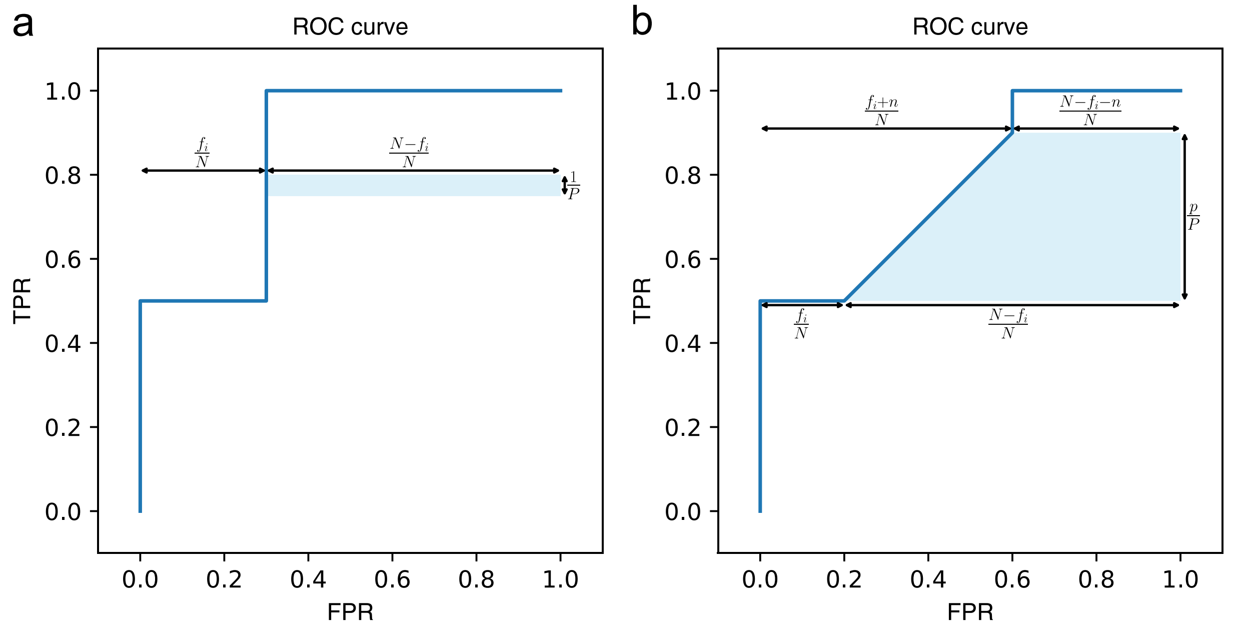

Fig. S25 Relationship between the Wilcoxon-Mann-Whitney statistic and AUROC. **a-b** The area gained when there are no ties (**a**) and there are ties (**b**) in the classification scores. All the symbols are defined in the section "Relationship between the Wilcoxon-Mann-Whitney statistic and AUROC" in Additional file 1: Supplementary Text.

Supplementary Tables

Tool	Lastest stable version	AUROC	AUPRC	Publication venue	Publication year	No. of citations	Estimated no. of uses (AUPRC, keywords 1)	Estimated no. of uses (AUPRC, keywords 2)	No. of uses (AUROC)
ROCR [14]	1.0-11	✓	✓	Bioinformatics	2005	3,316	116	302	2,590
Weka [22]	3.8	✓	✓	ACM SIGKDD Explorations Newsletter	2009	24,776	227	1,180	3,790
scikit-learn [15]	1.3.0	✓	✓	Journal of Machine Learning Research	2011	80,296	2,130	5,420	16,100
PerfMeas [16]	1.2.5	✓	✓	CRAN Package*	2014	13	8	10	8
PRROC [17]	1.3.1	✓	✓	Bioinformatics	2015	306	189	252	254
TensorFlow [21]	2.13.0	✓	✓	arXiv preprint	2016	27,612	430	1,010	3,060
precrec [20]	0.14.2	✓	✓	Bioinformatics	2017	186	86	149	146
TorchEval [23]**	0.0.6	✓	✓	Advances in Neural Information Processing Systems	2019	33,345	0	0	0
MLeval [18]	0.3	✓	✓	CRAN Package*	2020	93	14	23	85
yardstick [19]	1.2.0	✓	✓	CRAN Package*	2021	107	13	26	66
pROC [52]	1.18.4	✓	—	BMC Bioinformatics	2011	9,360	—	—	8,120
plotROC [53]	2.3.0	✓	—	Journal of Statistical Software	2017	170	—	—	167

Table S1 List of tools evaluated in this study. The 10 tools that can compute AUPRC are listed first, followed by 2 tools that can only compute AUROC but not AUPRC. In each category, the tools are ordered by year of corresponding publications. *For the CRAN packages, there were no associated formal papers available, leading to some publications where these packages were used without formal citation, making it difficult for Google Scholar to accurately count their citations. To resolve this problem, we conducted a manual search for each package name on Google Scholar and verified whether the resulting papers cited these packages. **TorchEval has been embedded into PyTorch officially (https://pytorch.org/torcheval/stable/) without a formal publication; we collected the number of citations of the original publication of PyTorch instead. As there is no stable version of TorchEval yet, we collected its latest version 0.0.6.

Issue	Can lead to overly-optimistic AUPRC		Can flip the order of two AUPRCs	
	With ties	Without ties	With ties	Without ties
①	Yes	No	Yes	No
②	Yes	No	Yes	No
③	No	No	Yes	Yes
④	Yes	No	Yes	No
⑤	Yes	Yes	Yes	Yes

Table S2 Consequences of the five issues in computing AUPRC.

Tool	ROC construction algorithm	AUROC calculation algorithm
ROCR	Connecting (FPR, TPR) anchor points	The trapezoidal rule (vertical)
Weka	Connecting (FPR, TPR) anchor points	The trapezoidal rule (horizontal)*
scikit-learn	Connecting (FPR, TPR) anchor points	The trapezoidal rule (vertical)
PerfMeas	No curve	The Wilcoxon-Mann-Whitney statistic
PRROC	Connecting (FPR, TPR) anchor points	The trapezoidal rule (vertical)
TensorFlow	No curve	The trapezoidal rule (vertical)**
precrec (default mode)	Connecting (FPR, TPR) anchor points including linearly interpolated points***	The trapezoidal rule (vertical)
precrec (aucroc mode)	No curve	The Wilcoxon-Mann-Whitney statistic
TorchEval	No curve	The trapezoidal rule (vertical)
MLeval	Connecting (FPR, TPR) anchor points	The trapezoidal rule (vertical)
yardstick	Connecting (FPR, TPR) anchor points	The trapezoidal rule (vertical)
pROC	Connecting $(1 - FPR, TPR)$ anchor points (The x-axis is reversed from 1 to 0)	The trapezoidal rule (vertical)
plotROC	Connecting (FPR, TPR) anchor points	The trapezoidal rule (vertical)

Table S3 Algorithms used by the 12 tools we surveyed to construct ROC and compute AUROC. *Weka states that it uses the Wilcoxon-Mann-Whitney statistic to compute AUROC in its source code. However, by checking the logic implemented by the source code and its naming scheme (i.e., the variable name is "area"), we believe it actually uses the trapezoidal rule (horizontal). **TensorFlow specifies the Riemann summation method in its source code when it computes AUROC, which is equivalent to the trapezoidal rule (vertical). ***Precrec employs linear interpolation with the interpolated points explicitly created to construct the ROC.

References

33. Keilwagen J, Grosse I, and Grau J. Area under precision-recall curves for weighted and unweighted data. PLoS ONE 2014; 9:e92209

34. Boyd K, Eng KH, and Page CD. Area under the precision-recall curve: point estimates and confidence intervals. *Machine Learning and Knowledge Discovery in Databases: European Conference, ECML PKDD 2013, Prague, Czech Republic, September 23-27, 2013, Proceedings, Part III 13*. Heidelberg, Germany: Springer, 2013 :451–66

35. Su W, Yuan Y, and Zhu M. A relationship between the average precision and the area under the ROC curve. *Proceedings of the 2015 International Conference on the Theory of Information Retrieval*. New York, NY, USA: Association for Computing Machinery, 2015 :349–52

36. Schütze H, Manning CD, and Raghavan P. Introduction to information retrieval. Vol. 39. Cambridge University Press Cambridge, 2008

37. Everingham M, Van Gool L, Williams CK, Winn J, and Zisserman A. The pascal visual object classes (VOC) challenge. International Journal of Computer Vision 2010; 88:303–38

38. Lupu M, Mayer K, Kando N, and Trippe AJ. Current challenges in patent information retrieval. Vol. 37. Heidelberg, Germany: Springer, 2017

39. Hirling D, Tasnadi E, Caicedo J, Caroprese MV, Sjögren R, Aubreville M, Koos K, and Horvath P. Segmentation metric misinterpretations in bioimage analysis. Nature Methods 2023 :1–4

40. Azzone M, Barucci E, Moncayo GG, and Marazzina D. A machine learning model for lapse prediction in life insurance contracts. Expert Systems with Applications 2022; 191:116261

41. He H and Ma Y. Imbalanced Learning: foundations, algorithms, and applications. NJ, USA: John Wiley & Sons, 2013

42. Chu Y, Zhang Y, Wang Q, Zhang L, Wang X, Wang Y, Salahub DR, Xu Q, Wang J, Jiang X, et al. A transformer-based model to predict peptide–HLA class I binding and optimize mutated peptides for vaccine design. Nature Machine Intelligence 2022; 4:300–11

43. Miller D, Stern A, and Burstein D. Deciphering microbial gene function using natural language processing. Nature Communications 2022; 13:5731

44. Schwab P, Mehrjou A, Parbhoo S, Celi LA, Hetzel J, Hofer M, Schölkopf B, and Bauer S. Real-time prediction of COVID-19 related mortality using electronic health records. Nature Communications 2021; 12:1058

45. Ye Q, Hsieh CY, Yang Z, Kang Y, Chen J, Cao D, He S, and Hou T. A unified drug–target interaction prediction framework based on knowledge graph and recommendation system. Nature Communications 2021; 12:6775

46. Majdandzic A, Rajesh C, and Koo PK. Correcting gradient-based interpretations of deep neural networks for genomics. Genome Biology 2023; 24:1–13

47. Wang W, Gao L, Ye Y, and Gao Y. CCIP: predicting CTCF-mediated chromatin loops with transitivity. Bioinformatics 2021; 37:4635–42

48. Fawcett T. An introduction to ROC analysis. Pattern Recognition Letters 2006; 27:861–74

49. Muschelli III J. ROC and AUC with a binary predictor: a potentially misleading metric. Journal of Classification 2020; 37:696–708

50. Hanley JA and McNeil BJ. The meaning and use of the area under a receiver operating characteristic (ROC) curve. Radiology 1982; 143:29–36

51. Cortes C and Mohri M. AUC optimization vs. error rate minimization. Advances in Neural Information Processing Systems 2003; 16:313–20

52. Robin X, Turck N, Hainard A, Tiberti N, Lisacek F, Sanchez JC, and Müller M. pROC: an open-source package for R and S+ to analyze and compare ROC curves. BMC Bioinformatics 2011; 12:1–8

53. Sachs MC. plotROC: a tool for plotting ROC curves. Journal of Statistical Software 2017; 79:1–19

54. Harrell Jr FE, Lee KL, and Mark DB. Multivariable prognostic models: issues in developing models, evaluating assumptions and adequacy, and measuring and reducing errors. Statistics in Medicine 1996; 15:361–87

55. Mason SJ and Graham NE. Areas beneath the relative operating characteristics (ROC) and relative operating levels (ROL) curves: statistical significance and interpretation. Quarterly Journal of the Royal Meteorological Society: A journal of the atmospheric sciences, applied meteorology and physical oceanography 2002; 128:2145–66
