# Supplementary material for: Commonly used software tools produce conflicting and overly-optimistic AUPRC values
Source: Genome Biol. 2024 May 13;25:118. doi: 10.1186/s13059-024-03266-y (PMC11089773; doi:10.1186/s13059-024-03266-y)
Supplement: Supplementary file 2 — Additional file 2. Review history. [file 13059_2024_3266_MOESM2_ESM.docx]

**1^st^ round**

**Reviewer 1**

In this manuscript, the authors perform a detailed benchmarking of ten tools for calculating and visualizing (the area under) precision-recall curves, and highlight differences between them as well as potential issues.

I found the manuscript nicely written and easy to follow, with lots of useful details provided in the supplementary notes. The evaluation is informative and technically sound. I have only relatively minor suggestions.

1. My main concern relates to the reproducibility of the results. While the methods section provides details on how the data sets were processed, I believe it would be very helpful (e.g., for future extensions and testing) to provide the final tables generated by the authors and supplied as input to the respective tools. The authors also write that they will set up a GitHub repository with the code they used, which I strongly agree with. The code submitted for the review contains a small example of how each package was called - while this is nice and useful to understand the interaction with the different tools, I would encourage the authors to consider whether, for reproducibility purposes, a more complete representation of the code they used to generate the results shown in the paper could be supplied.

2. In the Supplementary text, section "Mathematical details of PRC and AUPRC - The non-linear interpolation formula", the text states that the interpolation methods interpolate additional point(s) between A and B "if m>1", where m is the number of entities with a classification score of t_B. Should this condition rather be "if TP_B-TP_A>1"? With the current formulation, according to the statement on line 36 on page S1, a negative number of points (TP_B-TP_A-1) might be interpolated.

3. In the Supplementary section "Detailed discussions on the individual tools", the use of italics and a final () when referring to functions is a bit inconsistent. It was not clear to me whether this carried any significance, or if they should all be consistent.

Typos:
- Page 9, line 15: ATlat -> ATlas
- Page S8, line 8: skleran -> sklearn

**Reviewer 2**

In this work the authors assess the value of area under the precision recall curve metrics used by a very large number of scientists in the field. The message of the manuscript is that depending on the choice of methodology in computing these metrics, the methods that are evaluated could be ranked differently. This difference, in turn, could confirm (or not) the corresponding biological findings.
The authors detail the current state of the art and highlight the issues with existing tools. Indeed, the issue they bring up plagues the field as a whole. This reviewer has run into the described problems many many times.
The manuscript is well written, concise and to the point. The figures confirm the problems raised.
The only comment that I have is that there should be some thought about how to resolve the existing problem or at least what steps to take to address it.
I note that this probably should be just some possible considerations, as extensive text to address this point is likely beyond the scope of this manuscript.

**Authors’ response**

Reviewer 1:

1.1.Overall comment

Reviewer Comment:

In this manuscript, the authors perform a detailed benchmarking of ten tools for

calculating and visualizing (the area under) precision-recall curves, and highlight

differences between them as well as potential issues.

I found the manuscript nicely written and easy to follow, with lots of useful details

provided in the supplementary notes. The evaluation is informative and technically

sound. I have only relatively minor suggestions.

Author Response:

We are glad that the referee agrees that our study is informative and technically sound,

and our manuscript is nicely written. We thank the referee for all the comments and

suggestions given in general.

———

1.2.Code and data availability

Reviewer Comment:

My main concern relates to the reproducibility of the results. While the methods section

provides details on how the data sets were processed, I believe it would be very helpful

(e.g., for future extensions and testing) to provide the final tables generated by the

authors and supplied as input to the respective tools. The authors also write that they

will set up a GitHub repository with the code they used, which I strongly agree with.

The code submitted for the review contains a small example of how each package was

called - while this is nice and useful to understand the interaction with the different

tools, I would encourage the authors to consider whether, for reproducibility purposes,

a more complete representation of the code they used to generate the results shown in

the paper could be supplied.

Author Response:

We have now set up the GitHub and Zenodo repositories that include the materials for

reproducing all the results in our paper:

1) We have now included all our code for AUPRC computation and PRC visualization

of all the 10 tools;

2) We have now included all the processed data used in our study.

We believe these efforts can ensure the reproducibility of our results.

Excerpt from revised manuscript:

Availability of code and data

Our code is written in Java, Python and R. The reproducible code and all the data used

in this paper are available at GitHub [31] and Zenodo [32].

…

[31] Chen W, Miao C, Zhang Z, Fung CSH, Wang R, Chen Y, et al. Commonly used

software tools produce conflicting and overly-optimistic AUPRC values. GitHub. 2024.

https://github.com/wychencuhk/AUPRC_project.

[32] Chen W, Miao C, Zhang Z, Fung CSH, Wang R, Chen Y, et al. Commonly used

software tools produce conflicting and overly-optimistic AUPRC values. Zenodo. 2024.

https://doi.org/10.5281/zenodo.11076192.

———

1.3.Inaccuracy of the description of a mathematical detail

Reviewer Comment:

In the Supplementary text, section "Mathematical details of PRC and AUPRC - The

non-linear interpolation formula", the text states that the interpolation methods

interpolate additional point(s) between A and B "if m>1", where m is the number of

entities with a classification score of t_B. Should this condition rather be "if TP_B-

TP_A>1"? With the current formulation, according to the statement on line 36 on page

S1, a negative number of points (TP_B-TP_A-1) might be interpolated.

Author Response:

We thank the referee for spotting this important inaccuracy and have revised the

description accordingly. Indeed, if TPB = TPA, TPB - TPA - 1 would be negative. In

that case, although there are ties in classification score, the entities involved are all

actual negatives. As a result, they all lie on the same vertical line of the PRC and

therefore no interpolation is required.

We have modified the interpolation condition to “if TPB - TPA>1” as suggested by the

referee.

Excerpt from revised manuscript:
If TPB − TPA > 1, the interpolation methods interpolate additional point(s) between A
and B.
———
1.4.Format inconsistency
Reviewer Comment:
In the Supplementary section "Detailed discussions on the individual tools", the use of
italics and a final () when referring to functions is a bit inconsistent. It was not clear to
me whether this carried any significance, or if they should all be consistent.
Author Response:
We thank the referee for pointing out this inconsistency. We have denoted all the
functions with a () and removed all the italics previously imposed on the functions. This
change has improved the readability of our manuscript.
Excerpt from revised manuscript:
(Please see the revised manuscript.)
———
1.5.Typos
Reviewer Comment:Typos:
- Page 9, line 15: ATlat -> ATlas
- Page S8, line 8: skleran -> sklearn
Author Response:
We thank the referee for spotting these typos and have corrected them.
Excerpt from revised manuscript:
In the first scenario, we downloaded CITE-seq data produced from COVID-19 patient
blood samples by the COVID-19 Multi-Omic Blood ATlas (COMBAT) consortium [25].
…
For the first option, if the sklearn.metrics.precision_recall_curve() function is used to
construct the PRC and then the sklearn.metrics.auc() function is used to compute
AUPRC, …

Reviewer 2:

2.1.Well-written manuscript, concise and to the point
Reviewer Comment:
In this work the authors assess the value of area under the precision recall curve
metrics used by a very large number of scientists in the field. The message of the
manuscript is that depending on the choice of methodology in computing these
metrics, the methods that are evaluated could be ranked differently. This difference, in
turn, could confirm (or not) the corresponding biological findings.
The authors detail the current state of the art and highlight the issues with existing
tools. Indeed, the issue they bring up plagues the field as a whole. This reviewer has
run into the described problems many many times.
The manuscript is well written, concise and to the point. The figures confirm the
problems raised.
Author Response:
We are glad that the referee finds our manuscript well-written, concise, and to the
point, and that our figures confirm the problems raised. We thank the referee for all the
comments and suggestions given in general.
———
2.2.Possible considerations to resolve the existing problem or at least what steps to
take to address it
Reviewer Comment:
The only comment that I have is that there should be some thought about how to
resolve the existing problem or at least what steps to take to address it.
I note that this probably should be just some possible considerations, as extensive text
to address this point is likely beyond the scope of this manuscript.
Author Response:

We thank the referee for raising this important question and for the concrete

suggestion to add some possible considerations to the text.

We have thus included a paragraph in the Conclusions section to provide some

considerations. We hope these points can offer additional insights to researchers to

better perform classifier evaluation.

Excerpt from revised manuscript:

To address the problems, it is crucial to use tools that are free of the bugs described

and avoid using the linear interpolation method (Table 1). It is also necessary to state

clearly in manuscripts both the tool used (with its version number) and the underlying

methods implemented by the tool for producing PRC and AUPRC. Whenever feasible,

the adoption of multiple tools that implement different methods (e.g., one based on

continuous expectation and one based on AP) is recommended, with comprehensive

reporting of all their results.
